# Substituted 2-[(2-Oxo-2*H*-[1,2,4]triazino [2,3-*c*]quinazolin-6-yl)thio]acetamides with Thiazole and Thiadiazole Fragments: Synthesis, Physicochemical Properties, Cytotoxicity, and Anticancer Activity

**DOI:** 10.3797/scipharm.1208-07

**Published:** 2012-10-04

**Authors:** Sergey I. Kovalenko, Inna S. Nosulenko, Alexey Yu. Voskoboynik, Galina G. Berest, Lyudmyla N. Antypenko, Alexey N. Antypenko, Andrey M. Katsev

**Affiliations:** 1 Zaporozhye State Medical University, 69035, Zaporozhye, Ukraine.; 2 Crimean State Medical University, 95006, Simferopol, Ukraine.

**Keywords:** [1,2,4]Triazino[2,3-*c*]quinazolines, Thiazoles, Thiadiazoles, Anticancer, SAR, Bioluminescence inhibition

## Abstract

The series of novel *N*-R-2-[(3-R-2-oxo-2*H*-[1,2,4]triazino[2,3-*c*]quinazolin-6-yl)thio]acetamides with thiazole and thiadiazole fragments in a molecule were obtained by alkylation of potassium salts **1.1–1.4** by *N*-hetaryl-2-chloroacetamides and by aminolysis of activated acids **2.1–2.4** with *N,N’*-carbonyldiimidazole (CDI). The structures of compounds were determined by IR, ^1^H NMR, MS, and EI-MS analysis. The results of cytotoxicity evaluated by the bioluminescence inhibition of bacterium *Photobacterium leiognathi*, Sh1 showed that the compounds have considerable cytotoxicity. The synthesized compounds were tested for anticancer activity in NCI against 60 cell lines. Among the highly active compounds **3.1**, **3.2**, and **6.5**, 2-[(3-methyl-2-oxo-2*H-*[1,2,4]triazino[2,3-*c*]quinazolin-6-yl)thio]-*N*-(1,3-thiazol-2-yl)acetamide (**3.1**) was found to be the most active anticancer agent against the cell lines of colon cancer (GI_50_ at 0.41–0.69 μM), melanoma (GI_50_ 0.48–13.50 μM), and ovarian cancer (GI_50_ 0.25–5.01 μM). The structure-activity relationship (SAR-analysis) was discussed.

## Introduction

Unknown 2-[(3-R-2-oxo-2*H*-[1,2,4]triazino[2,3-*c*]quinazolin-6-yl)thio]acetic acids and their amides were identified as compounds with antibacterial and antitumor activities as the result of chemistry and pharmacology researches [1–4]. An important direction of further improvement of antitumor activity and expansion of the spectrum among such derivatives is a functionalization of amino substituents. We assume that the combination in one molecule *as*-triazino[2,3-*c*]quinazoline with thiazole, benzothiazole, and thiadiazole fragments, especially in the view of recent chemotherapy capacity, is one of the promising areas in the search for biologically active compounds with cytotoxic effects. Furthermore, that for these azoles and their derivatives, antibacterial [7, 9, 10, 13, 17, 24], antifungal [10, 13], antituberculous [6, 10], anticancer [5, 8, 15, 16, 18, 19, 21, 22], and other [11, 12, 14, 17, 20, 23, 31] activities are characteristic. So the introduction of «biophore» azole fragments in the structure of the «rigid» condensed heterocyclic system of *as-*triazino[2,3-*c*]quinazoline will likely lead to the change in direction and amplification of antitumor activity.

## Results and Discussion

### Chemistry

We selected the method of alkylation of potassium salts of 3-R-6-thio-6,7-dihydro-2*H-*[1,2,4]triazino[2,3-c]quinazolin-2-ones (**1.1–1.4**) by N-heteryl-2-chloroacetamides in dioxane or a dioxane-water mixture (2:1) due to the relatively low nucleophilic properties of the amino group of electron-deficient heterocycles (4-R-thiazolyl-2-, 6-R-1,3-benzothiazolyl-2-, 5-R-1,3,4-thiadiazolyl-2-amines) for the synthesis of N-(4-R-thiazolyl-2-, 6-R-1,3-benzothiazol-2-yl, 5-R-1,3,4-thiazolyl-2-(3-R-2-oxo-2*H*-[1,2,4]triazino[2,3-*c*]quinazolin-6-ylthio)acetamides (**3.1–3.6**, **4.1–4.11**, **5.1–5.9**) (Sch. 1). The reaction has a short duration (60–90 minutes), a high yield of products (73.5–99.3%), and a very high purity of the final reaction products. But as it turned out, the carbonyldiimidazole method of synthesis was thought that the corresponding acids **2.1–2.4** could be realized in the case of amides **3.1–3.3, 4.1, 4.2, 4.4–4.6, 4.9–4.11** (Sch. 1). The reaction was carried out by heating in DMF within 5–6 hours, but with resulting lower yields of the final products (62.8–54.3%). Moreover, the synthesized amides **3.1–3.3**, **4.1**, **4.2**, **4.4–4.6**, **4.9–4.11** required additional crystallization.

The conducted preliminary studies of the antitumor activity permitted the obtainment of substantiated evidence of its presence among the relevant *N*-2-[(3-R-2-oxo-2*H-*[1,2,4]triazino[2,3-*c*]quinazolin-6-yl)thio]acetamides and it is important that these derivatives could be regarded as a structure of «potential» pharmacophore aniline fragments in this heterocyclic system [3]. Therefore, agents of combined 2-[(5-amino-1,3,4-thiadizol-2-yl)thio]-*N*-phenylacetamide and 2-[(3-R-2-oxo-2*H*-[1,2,4]triazino[2,3-*c*]quinazolin-6-yl)thio]acetamide fragments, in our opinion, may become one of the most promising groups of organic compounds with antitumor activity. The relevant *N*-{5-[(2-(R-anilino)-2-oxoethyl)thio]-1,3,4-thiadiazole-2-yl}-2-chloroacetamides were synthesized by known methods, such as alkylation of 5-amino-1,3,4-thiadiazol-2-thiol by *N*-aryl-2-chloroacetamides in methanol, in the presence of sodium methoxide, followed by acylation of the formed products by 2-chloroacetyl chloride in dioxane in the presence of triethylamine. Alkylation of potassium salts **1.1** and **1.2** by *N*-{5-[(2-(R-anilino)-2-oxoethyl)thio]-1,3,4-thiadiazol-2-yl}-2-chloroacetamides was carried out in dioxane, refluxing for 90–120 min (Sch. 2), forming individual compounds with high yields **6.1–6.5** 67.7–94.8%.

The identity of the synthesized compounds was confirmed by LC-MS (APCI), elemental analysis, IR, ^1^H, ^13^C NMR, and MS. The chromato-mass study of the synthesized compounds **3.1–3.6**, **4.1–4.11**, **5.1–5.9**, **6.1–6.5** in a “soft” ionization (chemical ionization at atmospheric pressure) allowed each in each case to register the molecular ion peak [M+1] in high intensity. In addition, the characteristic ion [M+3] was present in the majority of compounds, which characterized the presence of the sulfur isotope in the molecule.

Characteristic vibrations of the ν_NH_-group at 3325–3008 cm^−1^, ν_CO_-group (bond «Amide I») at 1841–1659 cm^−1^, and the bond of combined stretchings and deformations of the N-H and C-N bonds («Amide II») at 1630–1556 cm^−1^ were observed in the IR spectra of compounds **3.1–3.6**, **4.1–4.11**, **5.1–5.9**, **6.1–6.5**. Vibrations of the carbonyl group in the triazinoquinazoline cycle appeared at 1684-1645 cm^−1^, and a characteristic outline of low intensity vibrations of the ν_-C=C-_ fragment of aromatic rings appeared at 1589–1468 cm^−1^, vibrations of the aromatic C-C bond at 1520 cm^−1^ and 1449 cm^−1^, out-of-plane vibrations γ_(=C-H)_ at 850–666 cm^−1^, and intensive vibrations at 2992–2829 cm^−1^, that are related to the symmetric and antisymmetric stretchings of the ν_CH3_- and ν_CH2_-groups.

The wide signal of the amide proton in the ^1^H NMR spectra for heterylamides **3.1–3.6**, **4.1–4.11**, **5.1–5.9** was observed in a weak field at 13.09–12.46 ppm. Two broad singlets of the proton of the NHCO-group of the relevant diamides **6.1–6.5** were recorded at 13.88–10.12 ppm and 13.88–13.14 ppm, the latter one was the most deshielded, located near the electron-deficient thiadiazole cycle. The two-proton singlet of the SCH_2_-group acetamides (**3.1–3.6**, **4.1–4.11**, **5.1–5.9**) was observed at 4.45–4.04 ppm and the protons of this functional group in diamides **6.1–6.5** appeared as singlets at 4.27–4.21 ppm and 4.38–4.34 ppm. The *as-*triazino[2,3-*c*]quinazoline system in the ^1^H-NMR spectra of compounds **3.1–3.6**, **4.1–4.11**, **5.1–5.9**, **6.1–6.5** was characterized by the subspectra signals of the protons ABCD-system: two doublet signals of the protons H-11 (8.50–8.38 ppm) and H-8 (7.62–7.21 ppm) and two triplet signals of the protons H-9 (7.94–7.83 ppm) and H-10 (7.70–7.30 ppm). It is important to note that the doublet H-8 and triplet H-9 mostly resonated together as a multiplet at 7.69–7.58 ppm and sometimes with aromatic protons of the substituent at position 3 (compounds **3.2**, **3.5**, **3.6**, **4.2**, **4.6**, **4.8**, **4.11**, **5.2**, **5.7**, **6.1**, **6.3**, **6.5**) or the heterocyclic fragment (compounds **3.5**, **3.6**, **5.2**, **5.3–5.5**). In the ^1^H-NMR spectra of amides **3.1–3.4** signals of the protons of the thiazole cycle resonated as doublets at 7.23–7.21 ppm (H-5) and 7.53–7.51 ppm (H-4). The singlet proton in position 5 of the thiadiazole system of amides **4.1–4.4** was observed at 9.18–8.82 ppm. In addition, the ^1^H-NMR spectra of the synthesized compounds was characterized by a set of signals of protons of aromatic substituents at position 4 of thiazole (compounds **3.5**, **3.6**), position 3 of the [1,2,4]triazino[2,3-*c*]quinazoline cycle (**3.2–3.6**, **4.2–4.4**, **4.6**, **4.8**, **4.9**, **4.11**, **5.2**, **5.4**, **5.5**, **5.7–5.9**, **6.1**, **6.3**, **6.5**), a set of signals of protons of aliphatic substituents at position 5 of thiadiazole (**4.5–4.11**) and position 6 of the 1,3-benzothiazole cycle (**5.1–5.5**). These protons, depending on the surroundings, have an appropriate multiplicity [3].

A «classical» chemical shift of carbon atoms of the -S-CH_2_-group in the ^13^C NMR spectra of amides **3.1**, **3.2**, **4.2**, **4.5–4.7**, **5.2**, **5.6,** and **5.7** was observed at 35.55–35.30 ppm. It is important that diamides **6.2**, **6.3** have two characteristic signals for the carbon atom of the -S-CH_2_-group at 35.28–35.23 ppm and 38.13–38.12 ppm. This aspect clearly confirms the S-regioselectivity of the reaction. In addition, the low magnetic field deshielded the carbon atom of the -CONH-group and carbons at the 2 and 6 positions of the [1,2,4]triazino[2,3-*c*]quinazoline cycle of amides (**3.1**, **3.2**, **4.2**, **4.5–4.7**, **5.2**, **5.6**, **5.7**, **6.2**, **6.3**) were present at 168.22–167.25 ppm, 164.77–158.45 ppm, and 161.02–154.59 ppm. Thus, the position and splitting of signals in the spectra of ^1^H and ^13^C NMR were in accordance with the proposed structures and clearly prove their structure and S-regioselectivity of alkylation.

For thiazolylamide **3.2,** low-intensity M^+•^ (3.0%) was observed in the mass spectra, the main directions of fragmentation were caused by the α- and β- break of the bond with the formation of fragmentary ions ^+•^ (F_1_) *m/z* 320 (2.7%), [Heteryl-S]^+•^ (F_2_) *m/z* 305 (8.4%), [CONHThiazolyl]^+•^ (F_3_) *m/z* 127 (20.7%), and [CH_2_CONHThiazolyl]^+•^ (F_4_) *m/z* 142 (23.2%), and the charge could be localized as on fragments F_1_, F_2,_ and also on F_3_ and F_4_. 5-R^1^-1,3,4-thiadiazolilamides **4.2**, **4.6**, **4.11** in the mass spectrum (EI) were also characterized by low intensity M^+^**^·^**^•^ (1.4–2.5%). It is important that for compounds **4.6**, **4.11** with donor substituents in position 5 of the 1,3,4-thiadiazole cycle, the main directions of fragmentation were caused by the break of the amino bond and *a*-rupture with the formation of the fragmentary ions [Heteryl-SCH_2_CO]^+•^ (F_1_) *m/z* 347 (5.4–8.5%) and [Heteryl-SCH_2_]^+•^ (F_2_) *m/z* 320 (7.6–20.2%). Meanwhile, the amide **4.2***a*-break was more typical with the formation of fragmented ions [Heteryl-SCH_2_]^+•^ (F_2_) *m/z* 320 (7.0%). In the mass spectra of 6-R^1^-1,3-benzothiazolylamides **5.2**, **5.6**, low-intensity M^+^**^·^**^•^ (1.2–1.7%) was also observed, but it is important that the fragmentation was similar to the corresponding arylamides. Thus, fragmentation of M^+•^ of compounds **5.2**, **5.6** was implemented by the break of the amino backbone with the formation of fragmentary ions [Heteryl-SCH_2_CO]^+^ (F_1_) with *m/z* 347 (17.6%) and *m/z* 285 (100.0%), which is probably associated with a significantly higher aromaticity of the benzothiazole substituent. It is important that the amides’ (**3.2**, **4.2**, **4.6**, **4.11**, **5.2**, **5.6**) ultimate fragmentation of ions [Heteryl-SCH_2_CO]^+•^, [Heteryl-SCH_2_]^+•^, [Heteryl-S]^+•^ was associated with the same process, namely, the degradation of the *as-*triazinoquinazoline system on the C(2)-C(3) and N(3)-N(4), and formation of highly stable ions with *m/z* 244 (9.10–100.0%), *m/z* 216 (100–50.1%), and *m/z* 203 (11.3–33.9%), respectively. The formed fragmentary ions eliminated fragments S, SCH_2_, CO, CNS, and CNO forming the ions *m/z* 187, 174, 170, 144, 143, and 142 of various intensities in the spectra.

In the mass spectra of diamide **6.2,** no molecular ion was observed as it is an unstable compound. The structure of diamide **6.2** was confirmed by the fragmentary ions [Heteryl-SCH_2_CO]^+•^ (F_1_) *m/z* 285 (22.5%), [HN-Thiadiazolyl-SCH_2_CO]^+•^ (F_2_) *m/z* 174 (40.3%), and [2-F-C_5_H_4_NH]^+•^ (F_2_) *m/z* 111 (100.0%). The fragmentation of the ion [Heteryl-SCH_2_CO]^+•^ (F_1_) was associated with the above process – the degradation of the *as-*triazinoquinazoline system on the C(2)-C(3) and N(3)-N(4) and the formation of the ion with *m/z* 244 (22.0%). The latter fragment eliminated the share of CO, CH_2_CO, S, CNO forming ions with *m/z* 217 (9.9%), 203 (40.7%), 171 (17.9%), 170 (21.3%), 129 (11.7%), 102 (42.7%).

### Pharmacology

#### Bioluminescence inhibition test

In the first stage, we researched the cytotoxicity of the synthesized compounds by the bioluminescent test of the luminescent bacteria *Photobacterium leiognathi* Sh1 (Table 1). Among the synthesized substances, compounds **3.1–3.6**, **4.1–4.11**, **5.1–5.9**, **6.1–6.5** on the model of acute action showed inhibiting properties at the concentration gradient of 0.025–0.25 mg/mL, which were typical for compounds **5.2–5.9** with benzothiazolamide and compounds **6.2–6.5**, **4.2**, **4.7**, **4.9–4.11** with the thiadiazolamide fragment in the molecule.

It’s important that among the mentioned compounds, only **4.10**, **5.3,** and **6.3** have shown high rates of inhibitory activity (inhibition of growth of 94.29–97.70%) at a concentration of 0.25 mg/mL in acute models of action. Meanwhile, in the chronic test, most of the synthesized compounds exhibited cytotoxic effects (Table 1). It should be noted that the compounds **3.1**, **3.4**, **3.5**, **4.2**, **4.4**, **4.6**, **5.1**, **5.5**, **5.7**, and **6.4** in the chronic test showed the effect of hormezis, inhibiting the intensity of bioluminescence in the smallest concentration (0.025 mg/mL). The increasing cytotoxicity of the compounds **3.1**, **3.3**, **3.5**, **3.6**, **4.2–4.6**, **4.10**, **5.1**, **5.3–5.6**, **5.8**, and **6.5** at a concentration of 0.01 and 0.25 mg/mL in the chronic test was expected.

The SAR study revealed that:

➢ [(3-R-2-oxo-2*H*-[1,2,4]triazino[2,3-*c*]quinazolin-6-yl)-thio]acetic acids with a thiazolamide fragment exhibited a high cytostatic effect against the luminescent bacteria *Photobacterium leiognathi* Sh1;
➢ a cytostatic effect of the synthesized compounds was retained with the replacement of thiazolamide by benzothiazole or thiadiazolamide fragments in the molecule,
➢ the replacement of the methyl group in position 3 of the triazino[2,3-*c*]quinazoline cycle by aryl function in most cases was the reason for the increased cytotoxic action,
➢ the replacement of the methyl group in position 6 in the benzothiazolamide fragment by the methoxygroup or chlorine didn’t lead to the loss of cytostatic action, and
➢ the introduction of a butyl substituent in position 5 of the thiadiazolamide fragment resulted in the initiation of growth of the luminescent bacteria *Photobacterium leiognathi* Sh1.

Thus, high levels of growth inhibition and cytotoxicity of compounds to the bacteria *P. leiognathi* Sh1 served as a prerequisite for further research on their antitumor activity.

#### Anticancer assay for preliminary in vitro testing

The synthesized *N*-R-2-[(3-R-2-oxo-2*H*-[1,2,4]triazino[2,3-*c*]quinazolin-6-yl)thio]acetamides with thiazole and thiadiazole fragments in the molecules (**3.1–3.6**, **4.1–4.11**, **5.1–5.9**, **6.1–6.5**) were submitted and evaluated at the single concentration of 10 μM in the panel of approximately 60 cancer cell lines. The human tumor cell lines were derived from nine different cancer types: leukemia, melanoma, lung, colon, CNS, ovarian, renal, prostate, and breast cancers. Primary anticancer assays were performed according to the US NCI protocol, which was described elsewhere (see e.g. http://dtp.nci.nih.gov) [29–31]. The results of primary screening are reported as the percent cancer cell line growth (GP%) and are presented in Table 2. The range of growth % shows the lowest and the highest growth % found among the different cancer cell lines.

The conducted screening studies of compounds at a concentration of 10 μM showed that the compounds **3.1**, **3.2**, **6.5** turned to be effective against most cancer cell lines (Table 2). Thus, *N*-(1,3-thiazol-2-yl)-2-[(3-methyl-2-oxo-2*H*-[1,2,4]triazino[2,3-*c*]quinazolin-6-yl)thio]-acetamide (**3.1**) showed high cytotoxic activity against 57 cell lines (inhibition of growth by 30–100%). Replacement of the methyl substituent at position 3 (compound **3.1**) of the triazinoquinazoline cycle by phenyl (**3.2**) and 4-methoxyphenyl (**3.4**) substituents led to a decrease in cytotoxicity against most cancer cell lines (Table 2). Modifications of the molecule by replacing the thiazole cycle (**3.1**, **3.2**, **3.4**) with thiadiazole (**4.1**, **4.2**, **4.4**, **4.9**) or benzothiazole (**5.1**, **5.4**, **5.7**, **5.8**) fragments caused a reduction in the spectrum and a decline in the antitumor activity. It is important that these compounds selectively inhibit the growth of the cell lines SSRF-CEM of leukemia. Further modification of the molecule by combining thiadiazole and arylamide fragments (compounds **6.4**, **6.5**) didn’t lead to significantly increased antitumor activity, but retained selective action against the cell line SSRF-CEM of leukemia and expanded the spectrum of compound **6.5** (inhibition of growth of 57 cell lines).

Further dose-dependent activity was investigated for the most effective compounds **3.1**, **3.2,** and **6.5** according to the standard NCI procedure for the 57–59 lines of nine types of cancer at five concentrations at 10-multiply dilution (100 μM, 10 μM, 1 μM, 0.1 μM, and 0.01 μM). In the experimental result, the three dose-dependent parameters were calculated: 1) GI_50_ – molar concentration of the compound that inhibits 50% net cell growth; 2) TGI – molar concentration of the compound leading to a total inhibition of cell growth; 3) LC_50_ – molar concentration of the compound leading to 50% net cell death. Furthermore, the mean graph midpoints (MG_MID) were calculated for each of the parameters, giving an average activity parameter over all of the cell lines for the tested compounds. For the calculation of the MG_MID, insensitive cell lines were included with the highest concentration tested (Table 3).

Among the studied substances, compound **3.1** showed the highest activity and broad spectrum with an average value of effective level of inhibition of growth of cell lines (MG_MID GI_50_ = 6.47 μM). The compound **3.1** showed high inhibition level against cell lines K-562 (GI_50_ = 0.34 μM) and SR (GI_50_ = 0.38 μM) of leukemia; cell lines NCI-H460 (GI_50_ = 0.41 μM) and NCI-H522 (GI_50_ = 0.22 μM) of NSC lung cancer; cell lines COLO 205 (GI_50_ = 0.45 μM), HCT-116 (GI_50_ = 0.44 μM), HCT-15 (GI_50_ = 0.69 μM), HT29 (GI_50_ = 0.41 μM), KM12 (GI_50_ = 0.49 μM) and SW-620 (GI_50_ = 0.54 μM) of colon cancer; cell lines MALME-3M (GI_50_ = 0.53 μM), M14 (GI_50_ = 0.56 μM), SK-MEL-5 (GI_50_ = 0.48 μM) and UACC-62 (GI_50_ = 0.58 μM) of melanoma; cell lines IGROV1 (GI_50_ = 0.81 μM), OVCAR-3 (GI_50_ = 0.26 μM) and NCI/ADR-RES (GI_50_ = 0.72 μM) of ovarian cancer; cell lines A498 (GI_50_ = 0.68 μM) and RXF 393 (GI_50_ = 0.69 μM) of renal cancer; cell lines MCF7 (GI_50_ = 0.48 μM), BT-549 (GI_50_ = 0.93 μM), and MDA-MB-468 (GI_50_ = 0.31 μM) of breast cancer. In addition, the apparent sensitivity of compounds **3.1** was revealed against almost all cell lines of colon cancer with GI_50_ = 0.41–0.69 μM, as well for cell lines NCI-H522 (GI_50_ = 0.22 μM, TI = 1.30 μM) and OVCAR-3 (GI_50_ = 0.26 μM, TI = 0.73 μM).

It should also be noted that among the average cytostatic effects, the most active substances turned out to be compounds **3.2** (MG_MID TI = 46.21 μM) and **6.5** (MG_MID TI = 55.96 μM). In addition, compound **3.2** revealed a high inhibition potential and cytostatic effect against the cell lines HOP-92 (GI_50_ = 2.04 μM, TI = 4.92 μM) of NSC lung cancer, SNB-75 (GI_50_ = 2.00 μM, TI = 4.47 μM) and OVCAR-4 (GI_50_ = 1.96 μM, TI = 4.05 μM) of ovarian cancer, and compound **6.5** - against the cell lines OVCAR-3 (GI_50_ = 2.19 μM, TI = 4.95 μM) and OVCAR-4 (GI_50_ = 2.11 μM, TI = 5.00 μM) of ovarian cancer.

The conducted research showed that compounds **3.1**, **3.2,** and **6.5** have an insignificant selectivity index (Table 4). Corresponding attention should be given to compound **3.1** with moderate selectivity index GI_50_ against ovarian cancer (SI = 3.16), compound **3.2** with moderate selectivity index GI_50_ against renal and prostate cancer (SI = 3.25; SI = 3.35, respectively), and compound **6.5** with moderate selectivity index GI_50_ against CNS cancer (SI = 3.35).

The SAR study revealed that:

➢ [(3-R-2-oxo-2*H*-[1,2,4]triazino[2,3-*c*]quinazolin-6-yl)-thio]acetic acids with a thiazolamide fragment demonstrated high antitumor activity,
➢ the replacement of thiazolamide by benzothiazole or thiadiazolamide fragments leed to a significant loss in antitumor activity,
➢ the replacement of the methyl group in position 3 of the triazino[2,3-*c*]quinazoline cycle by the phenyl, 4-methylphenyl, or 4-methoxyphenyl group caused the modest increase in activity against some cancer cell lines,
➢ the replacement of the methyl group at position 6 of the benzothiazolamide fragment by methoxygroup or chlorine didn’t lead to a significant increase in activity,
➢ the introduction of the butyl substituent in position 5 of the thiadiazolamide fragment was the reason for a slight increase in activity against some cancer cell lines, and
➢ the combination of one molecule of 2-[(5-amino-1,3,4-thiadiazol-2-yl)thio]-*N-*phenylacetamide and 2-[(3-R-2-oxo-2*H*-[1,2,4]triazino[2,3-*c*]quinazolin-6-yl)thio]-acetamide fragments leed to a high level of antitumor activity and requires further study.

## Conclusion

In the present paper, 31 new *N*-R-2-[(3-R-2-oxo-2*H*-[1,2,4]triazino[2,3-*c*]quinazolin-6-yl)thio]acetamides with thiazole and thiadiazole fragments in the molecules were described. The results of cytotoxicity evaluated by bioluminescence inhibition of the bacterium *Photobacterium leiognathi,* Sh1 showed that the compounds revealed significant cytotoxicity. Thirteen of the synthesized compounds were tested and most of them displayed antitumor activity against leukemia, melanoma, lung, colon, CNS, ovarian, renal, prostate, and breast cancers cell lines. Screening of anticancer activity *in vitro* yielded the most active compounds **3.1**, **3.2**, and **6.5** in micromolar concentrations at the GI_50_ level (GI_50_ mean graph midpoint varies from 6.47 up to 19.69). Compound **3.1** possessed the highest anticancer activity against the cell lines of colon cancer (GI_50_ 0.45–0.69 μM), melanoma (GI_50_ 0.48–13.50 μM), and ovarian cancer (GI_50_ 0.25–5.01 μM). The further modification of 2-[(3-R-2-oxo-2*H*-[1,2,4]triazino[2,3-*c*]quinazolin-6-yl)thio]acetic acid by broadening the base of amides is indisputably interesting and is being considered, which will be the premise for the creation of more effective anticancer agents among the barely known heterocyclic system.

## Experimental

### Chemistry

Melting points were determined in open capillary tubes and were uncorrected. The elemental analyses (C, H, N, S) were performed using the ELEMENTAR vario EL cube analyzer (USA). Analyses were indicated by the symbols of the elements or functions within ±0.3% of the theoretical values. The IR spectra (4000–600 cm^−1^) were recorded on a Bruker ALPHA FT-IR spectrometer (Bruker Bioscience, Germany) using a module for measuring attenuated total reflection (ATR). The ^1^H NMR spectra (400 MHz) and ^13^C NMR spectra (100 MHz): were recorded on the Varian-Mercury 400 (Varian Inc., Palo Alto, CA, USA) spectrometer with TMS as the internal standard in DMSO-*d_6_* solution. LC-MS were recorded using the chromatography / mass spectrometric system which consists of high performance liquid chromatograph «Agilent 1100 Series» (Agilent, Palo Alto, CA, USA) equipped with a diode-matrix and mass-selective detector «Agilent LC/MSD SL» (atmospheric pressure chemical ionization – APCI). The electron impact mass spectra (EI-MS) were recorded on the Varian 1200 L instrument at 70 eV (Varian, USA). The purity of all obtained compounds was checked by ^1^H-NMR and LC-MS.

Substances **1.1–1.4** and **2.1–2.4** were synthesized according to the reported procedures [1–3]. Other starting materials and solvents were obtained from commercially available sources and used without additional purification.

### General procedure for the synthesis of *N*-(4-R^1^-1,3-thiazolyl-2-)-2-[(3-R-2-oxo-2*H-*[1,2,4]triazino[2,3-*c*]quinazolin-6-yl)thio]acetamides (3.1–3.6)

#### Method A

*N*-(4-R-1,3-thiazol-2-yl)-2-chloroacetamide (0.011 mol) was added to a suspension of potassium salt of 3-R-6-thio-6,7-dihydro-2*H*-[1,2,4]triazino[2,3-*c*]quinazolin-2-ones (**2.1–2.4**) (0.01 mol) in 20 ml of dioxane or water-dioxane (1:2) and refluxed for 60–90 minutes. The mixture was cooled and poured into the water. The precipitate was filtered, dried, and recrystallized from the DMF-water (1:1).

#### Method B

*N,N*’-carbonyldiimidazole (1.95 g, 0.011 mol) was added to a solution of proper acid (**2.1–2.4**) (0.01 mol) in 10 ml of anhydrous DMF and heated in the water bath at 60–80°C for 1 hour with a calcium chloride tube. The proper 4-R-1,3-thiazolyl-2-amines (0.01 mol) were added with stirring to the resulting mixture and refluxed for 5–6 hours. The mixture was poured into the water, and neutralized to pH 6–7 by acetic acid. The precipitate was filtered, dried, and recrystallized from the DMF–water (1:1).

#### 2-[(3-Methyl-2-oxo-2H-[1,2,4]triazino[2,3-c]quinazolin-6-yl)thio]- N-(1,3-thiazol-2-yl)acetamide (**3.1**)

Yield: Method A, 79.3%; Method B, 61.3%; M.p. 280–282°C; IR (cm^−1^): 3166, 3121, 3093, 3068, 2954, 2910, 2820, 2726, 1678, 1668, 1590, 1559, 1512, 1469, 1425, 1376, 1359, 1328, 1282, 1261, 1206, 1177, 1136, 1120, 1056, 1042, 952, 873, 849, 804, 773, 733, 712, 701, 684, 629; ^1^H-NMR (400 MHz) δ: 2.41 (s, 3H, CH_3_), 4.33 (s, 2H, -S-C*H*_2_), 7.21 (d, 1H, *J=*3.6, H-5’ Thiazol), 7.51 (d, 1H, *J=*3.6, H- 4’ Thiazol), 7.60 (d, 1H, *J=*7.9, H-8), 7.65 (t, 1H, *J=*7.7, H-10), 7.92 (t, 1H, *J=*7.7, H-9), 8.45 (d, 1H, *J=*7.9, H-11), 12.47 (s, 1H, -C(O)NH); ^13^C-NMR (100 MHz) δ: 18.19 (CH_3_), 35.30 (-SCH_2_-), 114.10 (5’ Thiazol), 118.60 (11a), 126.00 (8), 126.55 (10), 128.01 (11), 135.99 (9), 138.27 (4’ Thiazol), 144.06 (3), 151.99 (11b), 154.32 (7a), 155.25 (6), 158.49 (2), 161.01 (2’ Thiazol), 166.85 (CONH); LC-MS (APCI), *m/z* = 385 [M+1]; Anal. calcd. for C_16_H_12_N_6_O_2_S_2_: C, 49.99; H, 3.15; N, 21.86; S, 16.68; Found: C, 49.99; H, 3.15; N, 21.86; S, 16.68.

#### 2-[(2-Oxo-3-phenyl-2H-[1,2,4]triazino[2,3-c]quinazolin-6-yl)thio]- N-(1,3-thiazol-2-yl)acetamide (**3.2**)

Yield: Method A, 83.9%; Method B, 58.2%; M.p. 264–266°C; IR (cm^−1^): 3173, 3091, 3063, 2921, 2793, 2691, 1696, 1667, 1583, 1563, 1555, 1503, 1486, 1467, 1442, 1396, 1370, 1342, 1326, 1314, 1282, 1260, 1239, 1162, 1132, 1074, 985, 965, 938, 873, 848, 812, 770, 755, 719, 694, 685, 651, 622; ^1^H-NMR (400 MHz) δ: 4.38 (s, 2H, -S-C*H*_2_), 7.21 (d, 1H, *J=*3.2, H-5’ Thiazol), 7.52 (d, 1H, *J=*3.2, H-4’ Thiazol), 7.69-7.58 (m, 5H, H-8, 10; H-3’, 4’, 5’ Ph), 7.94 (t, 1H, *J=*7.7, H-9), 8.31 (d, 2H, *J=*7.1, H-2’, 6’ Ph), 8.48 (d, 1H, *J=*7.9, H-11), 12.48 (s, 1H, -C(O)NH); ^13^C-NMR (100 MHz) δ: 35.32 (-SCH_2_), 114.14 (5’ Thiazol), 118.36 (11a), 126.12 (8), 126.69 (10), 128.15 (11), 128.96 (3’, 4’, 5’ Ph), 129.83 (2’, 6’ Ph), 132.08 (1’ Ph), 136.09 (9), 138.28 (4’ Thiazol), 144.09 (3), 149.70 (11b), 151.07 (7a), 154.59 (6), 158.45 (2), 160.13 (2’ Thiazol), 166.81 (CONH); EI-MS, *m/z* (I_rel_, %) = 446 (3.0), 373 (6.7), 305 (8.4), 293 (6.4), 269 (9.4), 246 (5.8), 245 (13.6), 244 (77.6), 243 (11.3), 218 (12.5), 217 (50.1), 216 (100.0), 214 (5.3), 213 (5.5), 204 (5.2), 203 (33.9), 188 (11.0), 187 (11.2), 186 (10.2), 185 (13.8), 174 (14.5), 172 (8.8), 171 (42.2), 170 (49.3), 148 (5.0), 145 (25.4), 144 (14.8), 143 (41.9), 142 (23.2), 140 (6.2), 127 (20.1), 126 (11.4), 116 (5.3), 113 (9.3), 104 (6.6), 103 (10.6), 90 (6.9), 88 (5.1), 77 (16.6), 76 (41.5), 75 (11.9), 73 (7.3), 63 (6.5), 60 (6.7), 58 (22.4), 57 (6.0), 55 (11.4), 51 (7.5), 50 (7.0), 47 (10.4), 46 (10.0), 45 (31.5), 44 (6.3); LC-MS (APCI), *m/z* = 447 [M+1], 449 [M+3]; Anal. calcd. for C_21_H_14_N_6_O_2_S_2_: C, 56.49; H, 3.16; N, 18.82; S, 14.36; Found: C, 56.49; H, 3.16; N, 18.82; S, 14.36.

#### 2-{[3-(4-Methylphenyl)-2-oxo-2H-[1,2,4]triazino[2,3-c]quinazolin-6-yl]thio}- N-(1,3-thiazol-2-yl)acetamide (**3.3**)

Yield: Method A, 81.4%; Method B, 54.8%; M.p. 274–275°C; IR (cm^−1^): 3243, 3183, 3115, 3066, 3008, 2980, 2942, 2867, 2802, 2700, 1683, 1660, 1626, 1583, 1546, 1514, 1485, 1469, 1395, 1366, 1328, 1263, 1236, 1172, 1155, 1137, 1104, 1066, 1021, 986, 971, 937, 905, 874, 851, 835, 818, 778, 765, 715, 701, 685, 677, 638, 624, 605; ^1^H-NMR (400 MHz) δ: 2.42 (s, 3H, 3-(4-CH_3_Ph)), 4.36 (s, 2H, -S-C*H*_2_), 7.23 (d, 1H, *J=*3.6, H-5’ Thiazol), 7.41 (d, 2H, *J=*8.3, H-3’, 4’ 3-(4-CH_3_Ph)), 7.52 (d, 1H, *J=*3.6, H- 4’ Thiazol), 7.61 (d, 1H, *J=*7.9, H-8), 7.67 (t, 1H, *J=*7.7, H-10), 7.94 (t, 1H, *J=*7.7, H-9), 8.25 (d, 2H, *J=*8.3, H-2’, 6’ 3-(4-CH3Ph)), 8.47 (d, 1H, *J=*7.9, H-11), 12.60 (s, 1H, -C(O)NH); LC-MS (APCI), *m/z* = 461 [M+1], 462 [M+2]; Anal. calcd. for C_22_H_16_N_6_O_2_S_2_: C, 57.38; H, 3.50; N, 18.25; S, 13.92; Found: C, 57.37; H, 3.49; N, 18.25; S, 13.90.

#### 2-{[3-(4-Methoxyphenyl)-2-oxo-2H-[1,2,4]triazino[2,3-c]quinazolin-6-yl]thio}- N-(1,3-thiazol-2-yl)acetamide (**3.4**)

Yield: Method A, 73.5%; M.p. 276–280°C; IR (cm^−1^): 3219, 3204, 3116, 3073, 3008, 2929, 2835, 2803, 2708, 1682, 1665, 1630, 1596, 1581, 1557, 1538, 1487, 1470, 1439, 1414, 1392, 1368, 1331, 1305, 1254, 1237, 1173, 1137, 1118, 1104, 1066, 1013, 986, 973, 938, 908, 873, 852, 819, 780, 763, 722, 697, 682, 624; ^1^H-NMR (400 MHz) δ: 3.87 (s, 3H, 4-CH_3_OPh), 4.37 (s, 2H, -S-C*H*_2_), 7.16 (d, 2H, *J=*8.8, H-3’, 4’ 4-CH_3_OPh), 7.23 (d, 1H, *J=*3.6, H-5’ Thiazol), 7.53 (d, 1H, *J=*3.6, H-4’ Thiazol), 7.62 (d, 1H, *J=*7.9, H-8), 7.66 (t, 1H, *J=*7.7, H-10), 7.93 (t, 1H, *J=*7.7, H-9), 8.38 (d, 2H, *J=*8.8, H-2’, 6’ 4-CH_3_OPh), 8.46 (d, 1H, *J=*7.9, H-11), 12.59 (s, 1H, -C(O)NH); LC-MS (APCI), *m/z* = 476 [M], 478 [M+2]; Anal. calcd. for C_22_H_16_N_6_O_3_S_2_: C, 55.45; H, 3.38; N, 17.64; S, 13.46; Found: C, 55.43; H, 3.36; N, 17.64; S, 13.45.

#### 2-[(2-Oxo-3-phenyl-2H-[1,2,4]triazino[2,3-c]quinazolin-6-yl)thio]-N-(4-phenyl-1,3-thiazol-2-yl)acetamide (**3.5**)

Yield: Method A, 88.5%; M.p. 265–267°C; IR (cm^−1^): 3584, 3172, 3057, 2972, 2927, 2866, 1692, 1666, 1651, 1626, 1586, 1565, 1550, 1503, 1484, 1470, 1443, 1369, 1322, 1311, 1286, 1267, 1247, 1183, 1156, 1137, 1105, 1072, 1061, 1026, 1001, 991, 942, 914, 904, 887, 873, 849, 813, 771, 756, 729, 718, 690, 673, 655; ^1^H-NMR (400 MHz) δ: 4.40 (s, 2H, -S-C*H*_2_-), 7.35 (t, 1H, *J=*7.5, H-4’ 4-Ph-Thiazol), 7.46 (t, 2H, *J=*7.5, H-3’, 5’4-Ph-Thiazol), 7.69-7.59 (m, 6H, H-3’, 4’, 5’ 3-Ph, H-5’4-Ph-Thiazol, H-8, 10), 7.97-7.91 (m, 3H, H-2’, 6’ 4-Ph-Thiazol, H-9), 8.31 (d, 2H, *J=*7.3, H-2’,6’3-Ph), 8.48 (d, 1H, *J=*7.9, H-11), 12.79 (s, 1H, - NHC(O)-); LC-MS (APCI), *m/z* = 522 [M], 524 [M+2]; Anal. calcd. for C_27_H_18_N_6_O_2_S_2_: C, 62.05; H, 3.47; N, 16.08; S, 12.27; Found: C, 62.05; H, 3.47; N, 16.08; S, 12.27.

#### 2-{[3-(4-Methoxyphenyl)-2-oxo-2H-[1,2,4]triazino[2,3-c]quinazolin-6-yl]thio}-N-(4-phenyl-1,3-thiazol-2-yl)acetamide (**3.6**)

Yield: Method A, 76.3%; M.p. 258–260°C; IR (cm^−1^): 3332, 3174, 3067, 2970, 2837, 1696, 1684, 1650, 1589, 1553, 1537, 1491, 1469, 1444, 1419, 1365, 1341, 1320, 1288, 1258, 1175, 1151, 1138, 1119, 1074, 1029, 990, 975, 941, 904, 841, 771, 763, 728, 716, 687, 657, 642, 613; ^1^H-NMR (400 MHz) δ: 3.87 (s, 3H, 3-(4-CH_3_OPh)), 4.39 (s, 2H, -S-C*H*_2_-), 7.17 (d, 2H, *J=*8.9, H-3’, 5’ 3-(4-CH_3_OPh)), 7.36 (t, 1H, *J=*7.5, H-4’ 4-Ph-Thiazol), 7.46 (t, 2H, *J=*7.5, H-3’, 5’ 4-Ph-Thiazol), 7.67-7.61 (m, 3H, H-5’4-Ph-Thiazol, H-8, 10), 7.96-7.89 (m, 3H, H-2’, 6’ 4-Ph-Thiazol, H-9), 8.39 (d, 2H, *J=*8.9, H-2’, 6’ 3-(4CH_3_OPh)), 8.46 (d, 1H, *J=*7.9, H-11), 12.79 (s, 1H, -NHC(O)-); LC-MS (APCI), *m/z* = 552 [M], 554 [M+2]; Anal. calcd. for C_28_H_20_N_6_O_2_S_2_: C, 60.86; H, 3.65; N, 15.21; S, 11.60; Found: C, 60.86; H, 3.65; N, 15.21; S, 11.60.

### General procedure for the synthesis of *N*-(5-R^1^-1,3,4-thiadiazol-2-yl)-2-[(3-R-2-oxo-2*H*-[1,2,4]triazino[2,3*-c*]quinazolin-6-yl)thio]acetamide (4.1–4.11)

#### Method A

*N*-(5-R^1^-1,3,4-thiadiazol-2-yl)-2-chloroacetamides (0.011 mol) were added to a suspension of potassium salt of 3-R-6-thio-6,7-dihydro-2*H*-[1,2,4]triazino[2,3-*c*]quinazolin-2-ones (**1.1–1.4**) (0.01 mol) in 20 ml of dioxane (**4.5**–**4.11**) or water-dioxane (1:2) (**4.1–4.4**) and refluxed for 60–90 minutes. The mixture was cooled and poured into the water. The precipitate was filtered, dried, and recrystallized from the DMF-water (1:1).

#### Method B

*N,N’-*carbonyldiimidazole (1.95 g, 0.011 mol) was added to a solution of proper acid (**2.1–2.4**) (0.01 mol) in 10 ml of anhydrous DMF and heated in the water bath at 60–80°C for 1 hour with a calcium chloride tube. The proper 5-R^1^-1,3,4-thiadiazolyl-2-amines (0.01 mol) were added with stirring to the resulting mixture and refluxed for 5–6 hours. The mixture was poured into the water, and neutralized to pH 6–7 by acetic acid. The precipitate was filtered, dried, and recrystallized from the DMF-water (1:1).

#### 2-[(3-Methyl-2-oxo-2H-[1,2,4]triazino[2,3-c]quinazolin-6-yl)thio]- N-(1,3,4-thiadiazol-2-yl)acetamide (**4.1**)

Yield: Method A, 80.4%; Method B, 61.7%; M.p. 306–309°C; IR (cm^−1^): 3153, 3104, 2905, 2826, 2711, 1701, 1659, 1587, 1556, 1509, 1466, 1434, 1382, 1365, 1323, 1285, 1261, 1214, 1190, 1160, 1129, 1103, 1045, 953, 906, 887, 872, 826, 805, 776, 717, 700, 686, 672, 630, 605; ^1^H-NMR (400 MHz) δ: 2.40 (t, 3H, 3*-*CH_3_), 4.37 (s, 2H, -S-C*H*_2_), 7.56 (d, 1H, *J=*7.9, H-8), 7.65 (t, 1H, *J=*7.7, H-10), 7.92 (t, 1H, *J=*7.7, H-9), 8.45 (d, 1H, *J=*7.9, H-11), 9.16 (s, 1H, H-5’ Thiadiazol), 12.98 (s, 1H, -C(O)NH); LC-MS (APCI), *m/z* = 386 [M+1], 388 [M+3]; Anal. calcd. for C_15_H_11_N_7_O_2_S_2_: C, 46.74; H, 2.88; N, 25.44; S, 16.64; Found: C, 46.72; H, 2.91; N, 25.47; S, 16.65.

#### 2-[(2-Oxo-3-phenyl-2H-[1,2,4]triazino[2,3-c]quinazolin-6-yl)thio]- N-(1,3,4-thiadiazol-2-yl)acetamide (**4.2**)

Yield: Method A, 89.4%; Method B, 53.3%; M.p. 295–297°C; IR (cm^−1^): 3173, 3055, 2952, 2904, 2838, 2728, 1706, 1675, 1595, 1566, 1554, 1510, 1488, 1470, 1446, 1358, 1326, 1315, 1287, 1270, 1242, 1183, 1168, 1139, 1119, 1103, 1081, 1050, 990, 959, 941, 903, 886, 872, 840, 812, 783, 771, 753, 710, 690, 653, 622; ^1^H-NMR (400 MHz) δ: 4.41 (s, 2H, -S-C*H*_2_), 7.69-7.57 (m, 5H, H-8, 10; H-3’, 4’, 5’ Ph), 7.94 (t, 1H, *J=*7.7, H-9), 8.29 (d, 2H, *J=*7.1, H-2’, 6’ Ph), 8.47 (d, 1H, *J=*7.9, H-11), 9.18 (s, 1H, H-5’ Thiadiazol), 13.09 (s, 1H, -C(O)NH); ^13^C-NMR (100 MHz) δ: 35.38 (-SCH_2_), 118.36 (11a), 126.14 (8), 126.63 (10), 128.21 (11), 128.96 (3’, 4’, 5’ Ph), 129.83 (2’, 6’ Ph), 132.08 (1’ Ph), 136.12 (9), 144.06 (5’ Thiadiazol), 149.33 (3), 149.76 (11b), 151.08 (7a), 154.53 (2’ Thiadiazol), 158.19 (6), 160.12 (2), 167.49 (CONH; EI-MS, *m/z* (I_rel_, %) = 447 (1.5), 320 (7.0), 305 (6.9), 271 (10.4), 244 (9.1), 243 (17.5), 218 (7.0), 217 (43.4), 216 (26.0), 215 (12.3), 203 (11.4), 189 (7.0), 188 (7.9), 187 (5.0), 186 (5.7), 185 (5.8), 171 (20.8), 170 (26.4), 161 (12.0), 160 (9.8), 148 (10.3), 145 (10.3), 144 (6.8), 143 (19.3), 142 (11.6), 134 (10.3), 130 (6.9), 129 (22.5), 128 (27.0), 104 (16.7), 103 (100.0), 102 (47.9), 101 (22.0), 91 (11.1), 90 (26.0), 88 (10.3), 77 (17.1), 76 (64.6), 75 (32.2), 74 (32.4), 73 (6.7), 72 (5.7), 70 (5.4), 69 (9.9), 64 (8.7), 63 (17.3), 62 (8.7), 61 (5.5), 60 (13.6), 59 (15.2), 58 (14.4), 57 (6.0), 56 (7.7), 47 (6.4), 46 (19.3), 45 (79.4), 44 (20.5), 43 (38.2), 42 (22.3), 41 (12.1); LC-MS (APCI), *m/z* = 448 [M+1], 449 [M+2]; Anal. calcd. for C_20_H_13_N_7_O_2_S_2_: C, 53.68; H, 2.93; N, 21.91; S, 14.33; Found: C, 53.68; H, 2.93; N, 21.91; S, 14.33.

#### 2-{[3-(4-Methylphenyl)-2-oxo-2H-[1,2,4]triazino[2,3-c]quinazolin-6-yl]thio}- N-(1,3,4-thiadiazol-2-yl)acetamide (**4.3**)

Yield: Method A, 84.5%; M.p. 292–294°C; IR (cm^−1^): 3170, 3057, 2952, 2899, 2831, 2728, 1705, 1672, 1594, 1565, 1551, 1504, 1468, 1450, 1413, 1361, 1328, 1308, 1285, 1272, 1240, 1183, 1169, 1152, 1138, 1118, 1104, 1074, 1051, 1020, 990, 959, 940, 906, 872, 831, 816, 782, 770, 714, 703, 685, 641, 628, 603; ^1^H-NMR (400 MHz) δ: 2.06 (s, 3H, 3-(4-C*H*_3_Ph)), 4.04 (s, 2H, -S-C*H*_2_), 7.05 (d, 2H, *J=*7.9, H-3’, 5’ 3-(4-C*H*_3_Ph)), 7.21 (d, 1H, *J=*7.9, H-8), 7.30 (t, 1H, *J=*7.7, H-10), 7.57 (t, 1H, *J=*7.7, H-9), 7.88 (d, 2H, *J=*7.9, H-2’, 6’ 3-(4-CH_3_Ph)), 8.45 (d, 1H, *J=*7.9, H-11), 8.82 (c, 1H, H-5’ Thiadiazol), 12.73 (s, 1H, -C(O)NH); LC-MS (APCI), *m/z* = 462 [M+1], 464 [M+3]; Anal. calcd. for C_21_H_15_N_7_O_2_S_2_: C, 54.65; H, 3.28; N, 21.24; S, 13.89; Found: C, 54.65; H, 3.28; N, 21.24; S, 13.89.

#### 2-{[3-(4-Methoxyphenyl)-2-oxo-2H-[1,2,4]triazino[2,3-c]quinazolin-6-yl]thio}- N-(1,3,4-thiadiazol-2-yl)acetamide (**4.4**)

Yield: Method A, 78.5%; Method B, 55.9%; M.p. 304–306°C; IR (cm^−1^): 3050, 3011, 2952, 2912, 2833, 2728, 1713, 1663, 1590, 1563, 1545, 1497, 1468, 1454, 1422, 1360, 1341, 1329, 1320, 1288, 1274, 1260, 1241, 1170, 1155, 1139, 1116, 1052, 1021, 992, 956, 941, 904, 872, 839, 818, 781, 768, 703, 685, 643, 623; ^1^H-NMR (400 MHz) δ: 3.87 (s, 3H, 3-(4-CH_3_OPh)), 4.40 (s, 2H, -S-C*H*_2_), 7.16 (d, 2H, *J=*8.7, H-3’, 5’ 3-(4-CH_3_OPh)), 7.55 (d, 1H, *J=*7.9, H-8), 7.66 (t, 1H, *J=*7.7, H-10), 7.93 (t, 1H, *J=*7.7, H-9), 8.38 (d, 2H, *J=*8.7, 2’, 6’ 3-(4-CH_3_OPh)), 8.46 (d, 1H, *J=*7.9, H-11), 9.17 (s, 1H, H-5’ Thiadiazol), 13.04 (s, 1H, -C(O)NH); LC-MS (APCI), *m/z* = 386 [M+1], 389 [M+3]; Anal. calcd. for C_21_H_15_N_7_O_3_S_2_: C, 52.82; H, 3.17; N, 20.53; S, 13.43; Found: C, 52.82; H, 3.17; N, 20.53; S, 13.43.

#### 2-[(3-Methyl-2-oxo-2H-[1,2,4]triazino[2,3-c]quinazolin-6-yl)thio]- N-(5-propyl-1,3,4-thiadiazol-2-yl)acetamide (**4.5**)

Yield: Method A, 90.1%; Method B, 59,3%; M.p. 247–252°C; IR (cm^−1^): 2962, 2910, 2835, 2727, 1703, 1664, 1587, 1557, 1508, 1469, 1426, 1360, 1336, 1284, 1265, 1240, 1198, 1183, 1169, 1137, 1120, 1108, 1044, 954, 873, 838, 778, 713, 701, 689, 674, 630, 613; ^1^H-NMR (400 MHz) δ: 0.92 (t, 3H, *J=*7.3, -(CH_2_)_2_-C*H_3_*), 1.70 (sext, 2H, *J^2^*=14.7, *J^3^*=7.3, - CH_2_-C*H**_2_-*CH_3_), 2.39 (s, 3H, 3-C*H*_3_), 2.94 (t, 2H, *J=*7.3, -C*H*_2_-CH_2_*-*CH_3_), 4.34 (s, 2H, -SC*H*_2_), 7.55 (d, 1H, *J=*7.9, H-8), 7.64 (t, 1H, *J=*7.7, H-10), 7.92 (t, 1H, *J=*7.7, H-9), 8.44 (d, 1H, *J=*7.9, H-11), 12.89 (s, 1H, -C(O)NH); ^13^C-NMR (100 MHz) δ: 13.70 (-CH_2_CH_2_*C*H_3_), 18.20 (3-CH_3_), 23.40 (-CH_2_*C*H_2_CH_3_), 32.65 (-*C*H_2_CH_2_CH_3_), 35.33 (-SCH_2_), 118.64 (11a), 126.02 (8), 126.51 (10), 128.06 (11), 136.03 (9), 144.03 (3), 151.98 (11b), 154.25 (2’ Thiadiazol), 155.28 (7a), 158.78 (5’ Thiadiazol), 161.02 (6), 164.51 (2), 167.28 (CONH); LC-MS (APCI), *m/z* = 428 [M+1], 430 [M+3]; Anal. calcd. for C_18_H_17_N_7_O_2_S_2_: C, 50.57; H, 4.01; N, 22.93; S, 15.00; Found: C, 50.57; H, 4.01; N, 22.93; S, 15.00.

#### 2-[(2-Oxo-3-phenyl-2H-[1,2,4]triazino[2,3-c]quinazolin-6-yl)thio]- N-(5-propyl-1,3,4-thiadiazol-2-yl)acetamide (**4.6**)

Yield: Method A, 93.9%; Method B, 54.3%; M.p. 244–246°C; IR (cm^−1^): 2964, 2868, 2727, 1696, 1671, 1589, 1562, 1509, 1488, 1468, 1445, 1381, 1337, 1312, 1283, 1269, 1241, 1172, 1136, 986, 965, 939, 873, 833, 811, 780, 767, 751, 706, 686, 652, 615; ^1^H-NMR (400 MHz) δ: 0.92 (t, 3H, *J=*7.3, -(CH_2_)_2_-C*H**_3_*), 1.70 (sext, 2H, *J^2^*=14.7, *J^3^*=7.3, -CH_2_-C*H**_2_*-CH_3_), 2.84 (t, 2H, *J=*7.3, -C*H*_2_-CH_2_*-*CH_3_), 4.38 (s, 2H, -S-C*H*_2_), 7.65-7.58 (m, 4H, H-3’, 4’, 5’ 3-Ph, H-8), 7.67 (t, 1H, *J=*7.7, H-10), 7.94 (t, 1H, *J=*7.7, H-9), 8.29 (d, 2H, *J=*7.3, 2’, 6’ 3-Ph), 8.48 (d, 1H, *J=*7.9, H-11), 12.96 (s, 1H, -C(O)NH); ^13^C-NMR (100 MHz) δ: 13.85 (-CH_2_CH_2_*C*H_3_), 22.98 (-CH_2_*C*H_2_CH_3_), 31.31 (-*C*H_2_CH_2_CH_3_), 35.32 (-SCH_2_), 118.40 (11a), 126.14 (8), 126.67 (10), 128.19 (4’ Ph, 11), 128.96 (3’, 5’ Ph), 129.82 (2’, 6’ Ph), 132.08 (1’ Ph), 136.11 (9), 144.06 (3), 149.76 (11b), 151.09 (2’ Thiadiazol), 154.53 (7a), 158.75 (5’ Thiadiazol), 6), 160.14 (6), 164.63 (2), 167.25 (CONH); EI-MS, *m/z* (I_rel_, %) = 489 (1.4), 353 (5.5), 347 (5.4), 320 (7.6), 313 (8.6), 293 (5.0), 284 (5.4), 244 (10.8), 219 (5.7), 218 (14.1), 217 (67.1), 216 (33.8), 215 (9.5), 203 (11.3), 191 (10.9), 190 (5.0), 189 (23.6), 188 (10.1), 187 (8.3), 186 (15.8), 185 (28.0), 171 (14.9), 170 (57.1), 161 (24.2), 160 (14.7), 159 (6.0), 157 (24.6), 156 (6.1), 154 (19.9), 148 (7.2), 145 (16.9), 144 (12.0), 143 (17.4), 142 (7.4), 141 (18.9), 134 (5.7), 131 (6.3), 130 (10.6), 129 (30.9), 128 (41.3), 127 (5.5), 117 (10.7), 116 (12.5), 115 (82.2), 114 (11.8), 104 (6.9), 103 (100.0), 101 (21.5), 101 (11.8), 100 (16.5), 99 (5.9), 97 (5.5), 95 (5.0), 91 (13.2), 90 (23.2), 89 (9.6), 88 (6.3), 87 (12.2), 86 (15.8), 85 (5.7), 84 (7.8), 77 (6.5), 76 (30.7), 74 (40.8), 73 (12.3), 72 (19.8), 71 (14.5), 70 (22.2), 69 (30.1), 68 (9.9), 67 (6.4), 66 (6.1), 65 (9.5), 64 (32.1), 63 (29.2), 62 (15.3), 61 (12.9), 60 (63.8), 59 (40.5), 58 (24.1), 57 (7.8), 56 (8.6), 55 (9.2), 54 (9.3), 53 (7.3), 52 (8.4), 51 (9.0), 47 (15.3), 46 (13.5), 45 (40.0), 43 (29.0), 42 (5.8), 41 (10.8), 40 (7.9); LC-MS (APCI), *m/z* = 490 [M+1], 492 [M+3]; Anal. calcd. for C_23_H_19_N_7_O_2_S_2_: C, 56.43; H, 3.91; N, 20.03; S, 13.10; Found: C, 56.43; H, 3.92; N, 20.04; S, 13.11.

#### N-(5-Butyl-1,3,4-thiadiazol-2-yl)-2-[(3-methyl-2-oxo-2H-[1,2,4]triazino[2,3-c]quinazolin-6-yl)thio]acetamide (**4.7**)

Yield: Method A, 73.6%; M.p. 234–236°C; IR (cm^−1^): 2956, 2924, 2857, 2738, 1690, 1671, 1589, 1557, 1508, 1488, 1468, 1447, 1383, 1330, 1314, 1285, 1271, 1243, 1168, 1134, 1102, 1002, 986, 969, 938, 877, 834, 810, 770, 753, 720, 705, 688, 653, 616; ^1^H-NMR (400 MHz) δ: 0.89 (t, 3H, *J=*7.3, -(CH_2_)_3_-C*H**_3_*), 1.33 (seks, 2H, *J*=7.3, -(CH_2_)_2_-C*H**_2_-*CH_3_), 1.66 (q, 2H, *J=*7.3, -CH_2_-C*H**_2_-*CH_2_*-*CH_3_), 2.39 (s, 3H, 3-C*H*_3_), 2.96 (t, 2H, *J=*7.3, -C*H*_2_(CH_2_)*_2_-*CH_3_), 4.33 (s, 2H, -S-C*H*_2_), 7.55 (d, 1H, *J=*7.9, H-8), 7.64 (t, 1H, *J=*7.7, H-10), 7.92 (t, 1H, *J=*7.7, H-9), 8.43 (d, 1H, *J=*7.9, H-11), 12.89 (s, 1H, -C(O)NH); ^13^C-NMR (100 MHz) δ: 14.00 (-CH_2_CH_2_CH_2_*C*H_3_), 18.17 (3-CH_3_), 21.96 (-CH_2_CH_2_*C*H_2_CH_3_), 29.06 (-CH_2_*C*H_2_CH_2_CH_3_), 31.60 (-*C*H_2_CH_2_CH_2_CH_3_), 35.34 (S-CH_2_), 118.62 (11a), 126.02 (8), 126.51 (10), 128.05 (11), 136.02 (9), 144.02 (3), 151.97 (11b), 154.22 (2’ Thiadiazol), 155.26 (7a), 158.75 (5’ Thiadiazol), 161.01 (6), 164.77 (2), 167.27 (CONH); LC-MS (APCI), *m/z* = 442 [M+1], 443 [M+2]; Anal. calcd. for C_19_H_19_N_7_O_2_S_2_: C, 51.69; H, 4.34; N, 22.21; S, 14.52; Found: C, 51.68; H, 4.33; N, 22.20; S, 14.51.

#### N-(5-Butyl-1,3,4-thiadiazol-2-yl)-2-[(2-oxo-3-phenyl-2H-[1,2,4]triazino[2,3-c]quinazolin-6-yl)thio]acetamide (**4.8**)

Yield: Method A, 89.4%; M.p. 266–270°C; IR (cm^−1^): 2956, 2924, 2857, 2738, 1690, 1671, 1589, 1557, 1508, 1488, 1468, 1447, 1383, 1330, 1314, 1285, 1271, 1243, 1168, 1134, 1102, 1002, 986, 969, 938, 877, 834, 810, 770, 753, 720, 705, 688, 653, 616; ^1^H-NMR (400 MHz) δ: 0.89 (t, 3H, *J=*7.3, -(CH_2_)_3_-C*H_3_*), 1.33 (sext, 2H, *J^2^*=14.9, *J^3^*=7.3, -(CH_2_)_2_-C*H_2_-*CH_3_), 1.66 (q, 2H, *J=*7.3, -CH_2_-C*H_2_-*CH_2_*-*CH_3_), 2.97 (t, 2H, *J=*7.3, -C*H*_2_-(CH_2_)*_2_-*CH_3_), 4.39 (s, 2H, -S-C*H*_2_), 7.69–7.58 (m, 5H, H-3’, 4’, 5’ 3-Ph, H-8, H-10), 7.94 (t, 1H, *J=*7.7, H-9), 8.29 (t, 2H, *J=*7.3, 2’, 6’ 3-Ph), 8.48 (d, 1H, *J=*7.9, H-11), 12.96 (s, 1H, -C(O)NH); LC-MS (APCI), *m/z* = 504 [M+1], 506 [M+3]; Anal. calcd. for C_24_H_21_N_7_O_2_S_2_: C, 57.24; H, 4.20; N, 19.47; S, 12.73; Found: C, 57.24; H, 4.20; N, 19.47; S, 12.71.

#### N-(5-Butyl-1,3,4-thiadiazol-2-yl)-2-{[3-(4-methylphenyl)-2-oxo-2H- [1,2,4]triazino[2,3-c]quinazolin-6-yl]thio}acetamide (**4.9**)

Yield: Method A, 74.4%; Method B, 56.8%; M.p. 262–265°C; IR (cm^−1^): 3244, 3078, 2955, 2914, 2856, 2725, 1696, 1674, 1624, 1609, 1588, 1565, 1548, 1515, 1503, 1486, 1468, 1417, 1376, 1331, 1309, 1274, 1241, 1184, 1165, 1137, 1106, 1072, 1021, 988, 966, 939, 902, 867, 856, 835, 826, 807, 780, 772, 755, 711, 701, 685, 626, 612; ^1^H-NMR (400 MHz) δ: 0.88 (t, 3H, *J=*7.3, -(CH_2_)_3_-C*H_3_*), 1.33 (sext, 2H, *J^2^*=14.9, *J^3^*=7.3, -(CH_2_)_2_-C*H_2_-*CH_3_), 1.66 (q, 2H, *J=*7.3, -CH_2_-C*H_2_-*CH_2_*-*CH_3_), 2.41 (s, 3H, 3-(4-C*H*_3_Ph)), 2.97 (t, 2H, *J=*7.3, -C*H*_2_-(CH_2_)*_2_-*CH_3_), 4.37 (s, 2H, -S-C*H*_2_), 7.40 (d, 2H, *J=*7.9, H-3’, 5’,3-(4-C*H*_3_Ph)), 7.58 (d, 1H, *J=*7.9, H-8), 7.66 (t, 1H, *J=*7.7, H-10), 7.93 (t, 1H, *J=*7.7, H-9), 8.24 (t, 2H, *J=*7.9, 2’, 6’ 3-(4-CH_3_Ph)), 8.46 (d, 1H, *J=*7.9, H-11), 12.92 (s, 1H, -C(O)NH); LC-MS (APCI), *m/z* = 518 [M+1], 520 [M+3]; Anal. calcd. for C_25_H_23_N_7_O_2_S_2_: C, 58.01; H, 4.48; N, 18.94; S, 12.39; Found: C, 57.98; H, 4.47; N, 18.97; S, 12.37.

#### N-(5-Isobutyl-1,3,4-thiadiazol-2-yl)-2-[(3-methyl-2-oxo-2H-[1,2,4]triazino[2,3-c]quinazolin-6-yl)thio]acetamide (**4.10**)

Yield: Method A, 56.6%; Method B, 59.8%; M.p. 262–264°C; IR (cm^−1^): 3066, 2961, 2920, 2859, 2712, 1692, 1662, 1622, 1589, 1563, 1503, 1468, 1423, 1382, 1361, 1325, 1283, 1259, 1205, 1166, 1133, 1120, 1043, 1018, 1001, 956, 872, 840, 780, 724, 687, 630, 612; ^1^H-NMR (400 MHz) δ: 0.91 (d, 6H, *J=*6.3, -CH_2_-CH(C*H_3_*)_2_), 1.97 (q, 1H, *J=*6.4, -CH_2_-C*H*(CH_3_)_2_), 2.39 (s, 3H, 3-C*H*_3_), 2.84 (d, 2H, *J=*6.9, -C*H_2_*-CH(CH*_3_*)_2_), 4.34 (s, 2H, -S-C*H*_2_), 7.54 (d, 1H, *J=*7.9, H-8), 7.64 (t, 1H, *J=*7.7, H-10), 7.91 (t, 1H, *J=*7.7, H-9), 8.43 (d, 1H, *J=*7.9, H-11), 12.94 (s, 1H, -C(O)NH); LC-MS (APCI), *m/z* = 442 [M+1], 444 [M+3]; Anal. calcd. for C_19_H_19_N_7_O_2_S_2_: C, 51.69; H, 4.34; N, 22.21; S, 14.52; Found: C, 51.71; H, 4.36; N, 22.23; S, 14.54.

#### N-(5-Isobutyl-1,3,4-thiadiazol-2-yl)-2-[(2-oxo-3-phenyl-2H-[1,2,4]triazino[2,3-c]quinazolin-6-yl)thio]acetamide (**4.11**)

Yield: Method A, 99.3%; Method B, 54.7%; M.p. 256–258°C; IR (cm^−1^): 3263, 3198, 3063, 2956, 2923, 2853, 1702, 1665, 1588, 1550, 1502, 1485, 1466, 1443, 1367, 1337, 1312, 1286, 1268, 1252, 1242, 1183, 1157, 1137, 1117, 1081, 1049, 1001, 989, 957, 940, 888, 870, 847, 812, 786, 773, 756, 692, 655, 614; ^1^H-NMR (400 MHz) δ: 0.92 (d, 6H, *J=*6.3, -CH_2_-CH(C*H_3_*)_2_), 1.99 (q, 1H, *J=*6.4, -CH_2_-C*H*(CH_3_)_2_), 2.84 (d, 2H, *J=*6.9, -C*H_2_*-CH(CH*_3_*)_2_), 4.38 (s, 2H, -S-C*H*_2_), 7.72-7.57 (m, 5H, H-3’, 4’, 5’ 3-Ph, H-8, H-10), 7.94 (t, 1H, *J=*7.7, H-9), 8.29 (t, 2H, *J=*7.3, 2’, 6’ 3-Ph), 8.48 (d, 1H, *J=*7.9, H-11), 12.94 (s, 1H, -C(O)NH); EI-MS, *m/z* (I_rel_, %) = 503 (2.5), 347 (8.5), 327 (13.9), 320 (20.2), 293 (8.2), 217 (16.7), 216 (6.0), 203 (21.5), 202 (7.6), 189 (9.9), 172 (5.2), 171 (26.2), 170 (31.9), 168 (10.9), 161 (6.0), 145 (9.6), 144 (8.3), 143 (25.0), 142 (29.2), 141 (48.5), 134 (7.1), 129 (11.8), 115 (23.6), 114 (6.5), 104 (14.6), 103 (100.0), 102 (25.9), 101 (9.9), 77 (14.3), 76 (52.4), 75 (28.4), 74 (21.9), 73 (11.4), 72 (10.9), 71 (8.4), 69 (11.7), 52 (7.2), 51 (13.6), 50 (17.0), 47 (10.6), 46 (9.9), 45 (22.6), 44 (16.5), 43 (86.0), 42 (16.8), 41 (52.0), 40 (5.9); LC-MS (APCI), *m/z* = 504 [M+1], 506 [M+3]; Anal. calcd. for C_24_H_21_N_7_O_2_S_2_: C, 57.24; H, 4.20; N, 19.47; S, 12.73; Found: C, 57.23; H, 4.19; N, 19.45; S, 12.70.

### General procedure for the synthesis of *N*-(6-R^1^-1,3-benzothiazol-2-yl)-2-[(3-R-2-oxo-2*H*-[1,2,4]triazino[2,3-*c*]quinazolin-6-yl)thio]acetamides (5.1–5.9)

*N*-(6-R^1^-1,3-benzothiazol-2-yl)-2-chloroacetamides (0.011 mol) were added to a suspension of potassium salt of 3-R-6-thio-6,7-dihydro-2*H*-[1,2,4]triazino[2,3-*c*]quinazolin-2-ones (**1.1–1.4**) (0.01 mol) in 20 ml of dioxane (**5.6**–**5.9**) or water–dioxane (1:2) (**5.1–5.5**) and refluxed for 60–90 minutes. The mixture was cooled and poured into the water. The precipitate was filtered, dried, and recrystallized from the DMF–water (1:1).

#### N-(6-Methyl-1,3-benzothiazol-2-yl)-2-[(3-methyl-2-oxo-2H-[1,2,4]triazino[2,3-c]quinazolin-6-yl)thio]acetamide (**5.1**)

Yield: 80.3%; M.p. 284–285°C; IR (cm^−1^): 3193, 3155, 3066, 2991, 2963, 2923, 2862, 1697, 1661, 1587, 1555, 1504, 1470, 1455, 1388, 1357, 1312, 1283, 1257, 1229, 1205, 1161, 1135, 1108, 1078, 1041, 989, 952, 898, 887, 868, 821, 803, 789, 777, 743, 699, 686, 672, 658, 630, 616; ^1^H-NMR (400 MHz) δ: 2.40 (s, 6H, 3-CH_3_; 6-CH_3_), 4.37 (s, 2H, -S-C*H*_2_), 7.27 (d, 1H, *J=*8.3, H-5’ Benzothiazol), 7.66-7.57 (m, 2H, H-8, 10), 7.88 (d, 1H, *J=*8.3, H-4’ Benzothiazol), 7.74 (s, 1H, H-7’ Benzothiazol), 7.90 (t, 1H, *J=*7.7, H-9), 8.44 (d, 1H, *J=*7.9, H-11), 12.73 (s, 1H, -C(O)NH); LC-MS (APCI), *m/z* = 450 [M+2]; Anal. calcd. for C_21_H_16_N_6_O_2_S_2_: C, 56.24; H, 3.60; N, 18.74; S, 14.30; Found: C, 56.24; H, 3.60; N, 18.75; S, 14.29.

#### N-(6-Methyl-1,3-benzothiazol-2-yl)-2-[(2-oxo-3-phenyl-2H-[1,2,4]triazino[2,3-c]quinazolin-6-yl)thio]acetamide (**5.2**)

Yield: Method A, 87.2%; M.p. 258–260°C; IR (cm^−1^): 3269, 3225, 3157, 3076, 2991, 2963, 2921, 2851, 1703, 1668, 1649, 1589, 1564, 1544, 1503, 1485, 1466, 1445, 1363, 1339, 1312, 1284, 1261, 1210, 1182, 1152, 1137, 1118, 1105, 1083, 1046, 1020, 1001, 989, 977, 940, 887, 873, 849, 809, 784, 773, 756, 692, 655, 615; ^1^H-NMR (400 MHz) δ: 2.40 (s, 3H, 6-CH_3_), 4.41 (s, 2H, -S-C*H*_2_), 7.27 (d, 1H, *J=*8.3, H-5’ Benzothiazole), 7.69–7.59 (m, 6H, H-3’, 4’, 5’ 3-Ph; H-4’ Benzothiazole; H-8, 10), 7.74 (s, 1H, H-7’ Benzothiazole), 7.92 (t, 1H, *J=*7.7, H-9), 8.31 (d, 2H, *J=*7.1, H-2’, 6’ 3-Ph), 8.47 (d, 1H, *J=*7.9, H-11), 12.77 (s, 1H, -C(O)NH); ^13^C-NMR (100 MHz) δ: 21.46 (CH_3_), 35.55 (-SCH_2_-), 118.36 (11a), 120.77 (4’ Benzothiazole), 121.79 (7’ Benzothiazole), 126.12 (8), 126.68 (10), 127.97 (11), 128.15 (4’ Ph), 128.95 (3’, 5’ Ph), 129.95 (2’, 6’ Ph), 132.07 (5’ Benzothiazole), 132.09 (7a’ Benzothiazole), 133.60 (1’ Ph), 136.10 (9), 147.05 (3), 147.08 (3a’ Benzothiazole), 149.74 (11b), 151.08 (2’ Benzothiazole), 154.60 (7a), 157.52 (6), 160.11 (2), 167.78 (CONH); EI-MS, *m/z* (I_rel_, %) = 510 (1.7), 348 (7.3), 347 (17.6), 334 (11.2), 333 (11.2), 306 (5.1), 245 (13.8), 244 (100.0), 242 (11.8), 218 (5.9), 217 (21.9), 216 (48.5), 205 (5.0), 204 (12.4), 203 (33.9), 191 (17.4), 190 (12.9), 189 (12.6), 186 (5.3), 185 (9.2), 177 (14.9), 175 (5.8), 174 (7.2), 172 (5.9), 171 (22.6), 170 (28.8), 165 (9.0), 164 (50.5), 163 (23.1), 161 (6.9), 150 (13.4), 149 (16.8), 148 (51.6), 147 (5.1), 145 (34.5), 144 (13.7) 143 (38.1), 142 (18.9), 137 (6.0), 136 (11.7), 129 (9.6), 122 (20.8), 121 (11.3), 119 (18.1), 118 (12.1), 117 (15.9), 116 (13.3), 110 (15.4), 109 (10.8), 104 (9.9), 103 (30.0), 102 (10.1), 92 (10.0), 91 (9.4), 90 (30.5), 89 (13.0), 86 (6.4), 84 (5.2), 83 (6.4), 82 (11.2), 77 (25.0), 76 (25.1), 75 (9.1), 69 (8.0), 65 (9.6), 64 (9.5), 63 (15.6), 60 (7.1), 58 (8.2), 57 (6.2), 56 (20.2), 53 (7.3), 52 (14.1), 51 (26.0), 50 (19.9), 45 (10.7), 43 (6.6); LC-MS (APCI), *m/z* = 511 [M+1], 513 [M+3]; Anal. calcd. for C_26_H_18_N_6_O_2_S_2_: C, 61.16; H, 3.55; N, 16.46; S, 12.56; Found: C, 61.18; H, 3.55; N, 16.47; S, 12.55.

#### N-(6-Methoxy-1,3-benzothiazol-2-yl)-2-[(3-methyl-2-oxo-2H-[1,2,4]triazino[2,3-c]quinazolin-6-yl)thio]acetamide (**5.3**)

Yield: 89.3%; M.p. 246–249°C; IR (cm^−1^): 3187, 3156, 3076, 2992, 2909, 2883, 2857, 2828, 1691, 1659, 1583, 1551, 1503, 1462, 1380, 1358, 1311, 1283, 1250, 1218, 1155, 1108, 1075, 1047, 1024, 981, 945, 893, 855, 823, 804, 776, 738, 690, 614; ^1^H-NMR (400 MHz) δ: 2.38 (s, 3H, 3-CH_3_), 3.79 (s, 3H, 6-CH_3_O), 4.36 (s, 2H, -S-C*H*_2_), 7.04 (d, 1H, *J=*8.5, H-5’ Benzothiazole), 7.52 (s, 1H, H-7’Benzothiazol), 7.73–7.57 (m, 6H, H-4’ Benzothiazole; H-8, 10), 7.90 (t, 1H, *J=*7.7, H-9), 8.43 (d, 1H, *J=*7.9, H-11), 12.65 (s, 1H, -C(O)NH); LC-MS (APCI), *m/z* = 465 [M+1], 466 [M+2]; Anal. calcd. for C_21_H_16_N_6_O_3_S_2_: C, 54.30; H, 3.47; N, 18.09; S, 13.80; Found: C, 54.31; H, 3.48; N, 18.09; S, 13.81.

#### N-(6-Methoxy-1,3-benzothiazol-2-yl)-2-[(2-oxo-3-phenyl-2H-[1,2,4]triazino[2,3-c]quinazolin-6-yl)thio]acetamide (**5.4**)

Yield: 89.6%; M.p. 271–273°C; IR (cm^−1^): 3272, 3240, 3067, 3002, 2952, 2913, 2888, 2855, 2830, 1702, 1666, 1611, 1590, 1565, 1546, 1503, 1485, 1461, 1435, 1414, 1365, 1338, 1324, 1303, 1290, 1265, 1242, 1229, 1210, 1179, 1143, 1112, 1080, 1065, 1028, 1001, 990, 976, 939, 887, 871, 854, 810, 788, 775, 755, 736, 692, 654, 612; ^1^H-NMR (400 MHz) δ: 3.82 (s, 3H, 6-CH_3_O), 4.43 (s, 2H, -S-C*H*_2_), 7.06 (d, 1H, *J^3^*=8.5, *J^4^*=2.2, H-5’ Benzothiazole), 7.52 (s, 1H, H-7’ Benzothiazole), 7.70–7.58 (m, 6H, H-4’ Benzothiazole; H-3’, 4’, 5’3-Ph; H-8, 10), 7.93 (t, 1H, *J=*7.7, H-9), 8.32 (d, 2H, *J=*7.3, H-2’, 6’ 3-Ph), 8.50 (d, 1H, *J=*7.9, H-11), 12.46 (s, 1H, -C(O)NH); LC-MS (APCI), *m/z* = 527 [M+1], 529 [M+3]; Anal. calcd. for C_26_H_18_N_6_O_3_S_2_: C, 59.30; H, 3.45; N, 15.96; S, 12.18; Found: C, 59.30; H, 3.43; N, 15.93; S, 12.18.

#### N-(6-Methoxy-1,3-benzothiazol-2-yl)-2-{[3-(4-methylphenyl)-2-oxo-2H- [1,2,4]triazino[2,3-c]quinazolin-6-yl]thio}acetamide (**5.5**)

Yield: A, 88.8%; M.p. 256–259°C; IR (cm^−1^): 3279, 3247, 3069, 3006, 2962, 2949, 2916, 2887, 2854, 1705, 1664, 1610, 1589, 1562, 1547, 1495, 1462, 1435, 1363, 1339, 1322, 1305, 1287, 1265, 1244, 1211, 1185, 1148, 1116, 1065, 1029, 994, 977, 941, 887, 872, 849, 834, 809, 794, 788, 774, 735, 714, 687, 645, 630, 609; ^1^H-NMR (400 MHz) δ: 2.42 (s, 3H, 3-(4-CH_3_Ph)), 3.80 (s, 3H, 6-CH_3_O), 4.40 (s, 2H, -S-C*H*_2_), 7.05 (d, 1H, *J^3^*=8.9, *J^4^*=2.3, H-5’ Benzothiazole), 7.41 (d, 2H, *J^3^*=7.9, H-3’, 5’ 3-(4-CH_3_Ph)), 7.54 (s, 1H, H-7’ Benzothiazole), 7.71-7.61 (m, 3H, H-4’ Benzothiazole, H-8, H-10), 7.89 (t, 1H, *J=*7.7, H-9), 8.25 (d, 2H, *J=*7.9, H-2’, 6’ 3-(4-CH_3_Ph)), 8.45 (d, 1H, *J=*7.9, H-11), 12.69 (s, 1H, -C(O)NH); LC-MS (APCI), *m/z* = 541 [M+1], 543 [M+3]; Anal. calcd. for C_27_H_20_N_6_O_3_S_2_: C, 59.99; H, 3.73; N, 15.54; S, 11.86; Found: C, 60.01; H, 3.77; N, 15.54; S, 11.88.

#### N-(6-Chloro-1,3-benzothiazol-2-yl)-2-[(3-methyl-2-oxo-2H-[1,2,4]triazino[2,3-c]quinazolin-6-yl)thio]acetamide (**5.6**)

Yield: 87.4%; M.p. 270–272°C; IR (cm^−1^): 3155, 3056, 2982, 2923, 2860, 1694, 1652, 1582, 1540, 1503, 1468, 1435, 1379, 1358, 1297, 1280, 1253, 1225, 1205, 1153, 1133, 1110, 1096, 1078, 1038, 986, 951, 892, 866, 822, 777, 759, 741, 698, 684, 629; ^1^H-NMR (400 MHz) δ: 2.42 (s, 3H, 3-CH_3_), 4.41 (s, 2H, -S-C*H*_2_), 7.45 (d, 1H, *J=*8.5, H-5’ Benzothiazol), 7.65-7.59 (m, 2H, H-8, 10), 7.77 (d, 1H, *J=*8.5, H-4’ Benzothiazol), 7.83 (t, 1H, *J=*7.7, H-9), 8.05 (s, 1H, H-7’ Benzothiazol), 8.45 (d, 1H, *J=*7.9, H-11), 12.69 (s, 1H, -C(O)NH); ^13^C-NMR (100 MHz) δ: 18.18 (CH_3_), 35.55 (-SCH_2_-), 118.61 (11a), 121.92 (4’ Benzothiazol), 122.34 (7’ Benzothiazol), 126.01 (8), 122.52 (6’ Benzothiazol), 126.99 (5’ Benzothiazol), 128.01 (10), 128.18 (11), 133.65 (7a’ Benzothiazol), 136.00 (9), 144.04 (3a’ Benzothiazol), 147.86 (3), 151.99 (11b), 154.24 (2’ Benzothiazol), 155.30 (7a), 159.26 (6), 160.98 (2), 168.22 (CONH); EI-MS, *m/z* (I_rel_, %) = 468 (1.2), 286 (14.2), 285 (100.0), 254 (5.5), 245 (8.9), 244 (43.0), 243 (6.6), 218 (6.7), 217 (24.1), 216 (55.0), 215 (5.7), 189 (7.0), 188 (8.1), 186 (13.3), 185 (8.9), 184 (25.7), 183 (6.5), 161 (11.0), 160 (10.2), 159 (5.3), 157 (10.2), 156 (8.2), 130 (11.7), 129 (28.5), 122 (7.2), 102 (21.0), 95 (5.7), 90 (11.4), 69 (12.3), 64 (7.3), 63 (11.2); LC-MS (APCI), *m/z* = 470 [M+2]; Anal. calcd. for C_20_H_13_ClN_6_O_2_S_2_: C, 51.23; H, 2.79; Cl, 7.56; N, 17.92; S, 13.67; Found: C, 51.21; H, 2.80; Cl, 7.56; N, 17.90; S, 13.65.

#### N-(6-Chloro-1,3-benzothiazol-2-yl)-2-[(2-oxo-3-phenyl-2H-[1,2,4]triazino[2,3-c]quinazolin-6-yl)thio]acetamide (**5.7**)

Yield: 90.4%; M.p. 247–249°C; IR (cm^−1^): 3259, 3222, 3066, 2989, 2964, 2908, 2886, 2852, 1703, 1667, 1591, 1566, 1537, 1503, 1485, 1468, 1443, 1398, 1363, 1339, 1311, 1300, 1287, 1260, 1241, 1182, 1152, 1137, 1116, 1097, 1082, 1050, 1020, 1000, 990, 977, 939, 887, 872, 810, 785, 772, 759, 733, 695, 655, 612; ^1^H-NMR (400 MHz) δ: 4.45 (s, 2H, -S-C*H*_2_), 7.47 (d, 1H, *J^3^*=8.6, *J^4^*=1.7, H-5’ Benzothiazole), 7.69-7.57 (m, 5H, H-8, 10; H-3’, 4’, 5’ Ph), 7.78 (d, 1H, *J=*8.6, H-4’ Benzothiazole), 7.92 (t, 1H, *J=*7.7, H-9), 8.06 (s, 1H, H-7’ Benzothiazole), 8.33 (d, 2H, *J=*7.3, H-2’, 6’ Ph), 8.50 (d, 1H, *J=*7.9, H-11), 12.72 (s, 1H, -C(O)NH); ^13^C-NMR (100 MHz) δ: 35.54 (-SCH_2_-), 118.40 (11a), 121.95 (4’ Benzothiazole), 122.36 (6’, 7’ Benzothiazole), 126.14 (5’ Benzothiazole), 126.66 (8), 127.00 (10), 128.19 (11, 4’ Ph), 128.96 (3’, 5’ Ph), 129.84 (2’, 6’ Ph), 132.08 (7a’ Benzothiazole), 133.66 (1’ Ph), 138.12 (9), 144.07 (3), 147.99 (3a’ Benzothiazole), 149.78 (11b), 151.09 (2’ Benzothiazole), 154.54 (7a), 159.26 (6), 160.12 (2), 168.19 (CONH); LC-MS (APCI), *m/z* = 530 [M-H], 532 [M+1], 534 [M+3]; Anal. calcd. for C_25_H_15_ClN_6_O_2_S_2_: C, 56.55; H, 2.85; Cl, 6.68; N, 15.83; S, 12.08; Found: C, 56.56; H, 2.87; Cl, 6.70; N, 15.85; S, 12.09.

#### N-(6-Chloro-1,3-benzothiazol-2-yl)-2-{[3-(4-methylphenyl)-2-oxo-2H-[1,2,4]triazino[2,3-c]quinazolin-6-yl]thio}acetamide (**5.8**)

Yield: 84.4%; M.p. 279–280°C; IR (cm^−1^): 3195, 3129, 3060, 2981, 2962, 2923, 2851, 1706, 1645, 1583, 1542, 1516, 1489, 1470, 1454, 1439, 1394, 1342, 1308, 1282, 1262, 1186, 1153, 1139, 1122, 1107, 1098, 1077, 1054, 1020, 983, 944, 884, 874, 835, 802, 771, 711, 687, 662, 643, 629, 612; ^1^H-NMR (400 MHz) δ: 2.48 (s, 3H, 3-(4-CH_3_Ph)), 4.43 (s, 2H, -S-C*H*_2_), 7.42 (d, 2H, *J=*8.1, H-3’, 5’ 3-(4-CH_3_Ph)); 7.48 (d, 1H, *J^3^*=8.5, *J^4^*=2.2, H-5’ Benzothiazole), 7.68-7.60 (m, 2H, H-8, 10), 7.80 (d, 1H, *J=*8.5, H-4’ Benzothiazole), 7.91 (t, 1H, *J=*7.7, H-9), 8.10 (s, 1H, H-7’ Benzothiazole), 8.25 (d, 2H, *J=*8.1, H-2’, 6’ 3-(4-CH_3_Ph)), 8.47 (d, 1H, *J=*7.9, H-11), 12.91 (s, 1H, -C(O)NH); Anal. calcd. for C_26_H_17_ClN_6_O_2_S_2_: C, 57.30; H, 3.14; Cl, 6.50; N, 15.42; S, 11.77; Found: C, 57.29; H, 3.16; Cl, 6.53; N, 15.42; S, 11.75.

#### N-(6-Chloro-1,3-benzothiazol-2-yl)-2-{[3-(4-methoxyphenyl)-2-oxo-2H- [1,2,4]triazino[2,3-c]quinazolin-6-yl]thio}acetamide (**5.9**)

Yield: 80.2%; M.p. 258–260°C; IR (cm^−1^): 3200, 3165, 3117, 3059, 2965, 2925, 2852, 1701, 1664, 1640, 1587, 1563, 1538, 1492, 1468, 1444, 1365, 1341, 1303, 1288, 1275, 1255, 1178, 1152, 1141, 1118, 1098, 1079, 1053, 1024, 993, 977, 941, 887, 867, 851, 825, 813, 784, 773, 762, 727, 707, 686, 638, 626, 615; ^1^H-NMR (400 MHz) δ: 3.91 (s, 3H, 3-(4-CH_3_OPh)), 4.45 (s, 2H, -S-C*H*_2_), 7.15 (d, 2H, *J=*8.9, H-3’, 5’ 3-(4-CH_3_OPh)); 7.46 (d, 1H, *J^3^*=8.5, *J^4^*=2.2, H-5’ Benzothiazole), 7.69-7.63 (m, 2H, H-8, 10), 7.77 (d, 1H, *J=*8.5, H-4’ Benzothiazole), 7.91 (t, 1H, *J=*7.7, H-9), 8.04 (s, 1H, H-7’ Benzothiazole), 8.40 (d, 2H, *J=*8.9, H-2’, 6’ 3-(4-CH_3_OPh)), 8.50 (d, 1H, *J=*7.9, H-11), 12.54 (s, 1H, -C(O)NH); LC-MS (APCI), *m/z* = 561 [M], 563 [M+2]; Anal. calcd. for C_26_H_17_ClN_6_O_3_S_2_: C, 55.66; H, 3.05; Cl, 6.32; N, 14.98; S, 11.43; Found: C, 55.66; H, 3.05; Cl, 6.31; N, 14.96; S, 11.42.

### General procedure for the synthesis of *N-(*5-{[2-(R^1^-anilino)-2-oxoethyl]thio}-1,3,4-thiadiazol-2-yl)-2-[(3-R-2-oxo-2*H*-[1,2,4]triazino[2,3-*c*]quinazolin-6-yl)thio]acetamides (6.1–6.5)

*N*-{5-[(2-(R-anilino)-2-oxoethyl)thio]-1,3,4-thiadiazol-2-yl}-2-chloroacetamides (0.011 mol) were added to a suspension of potassium salt of 3-R-6-thio-6,7-dihydro-2*H-*[1,2,4]triazino[2,3-*c*]quinazolin-2-ones (**2.1–2.2**) (0.01 mol) in 20 ml of dioxane and refluxed for 60–90 minutes. The mixture was cooled and poured into the water. The precipitate was filtered, dried, and recrystallized from DMF–water (1:1).

#### 4-[({[5-({[(2-Oxo-3-phenyl-2H-[1,2,4]triazino[2,3-c]quinazolin-6-yl)thio]acetyl}amino)- 1,3,4-thiadiazol-2-yl]thio}acetyl)amino]benzoic acid (**6.1**)

Yield: 67.7%; M.p. 214–216°C; IR (cm^−1^): 3314, 3265, 3118, 3073, 2924, 2445, 1841, 1673, 1620, 1598, 1537, 1513, 1504, 1484, 1444, 1393, 1326, 1312, 1271, 1242, 1185, 1167, 1127, 1108, 1076, 1041, 1009, 995, 965, 941, 863, 843, 812, 769, 750, 717, 686, 654, 631, 611; LC-MS (APCI), *m/z* = 660 [M+4]; Anal. calcd. for C_29_H_20_N_8_O_5_S_3_: C, 53.04; H, 3.07; N, 17.06; S, 14.65; Found: C, 53.04; H, 3.07; N, 17.06; S, 14.65.

#### N-[5-({2-[(2-Fluorophenyl)amino]-2-oxoethyl}thio)-1,3,4-thiadiazol-2-yl]-2-[(3-methyl-2-oxo-2H-[1,2,4]triazino[2,3-c]quinazolin-6-yl)thio]acetamide (**6.2**)

Yield: 72.1%; M.p. 248–251°C; IR (cm^−1^): 3325, 2978, 2921, 2852, 1712, 1668, 1655, 1585, 1562, 1527, 1504, 1469, 1453, 1402, 1370, 1330, 1317, 1285, 1265, 1253, 1211, 1191, 1161, 1145, 1103, 1059, 1047, 1009, 956, 887, 805, 783, 751, 717, 700, 683, 663, 633, 606; ^1^H-NMR (400 MHz) δ: 2.39 (s, 3H, 3-C*H*_3_), 4.26 (s, 2H, -S-C*H*_2_-C(O)NHPh-F-2), 4.34 (s, 2H, -S-C*H*_2_-), 7.18-7.12 (m, 2H, H-3’, 6’ 2-FPh), 7.25 (t, 1H, *J=*7.3, H-5’ 2-F-Ph), 7.55 (d, 1H, *J=*7.9, H-8), 7.63 (t, 1H, *J=*7.7, H-10), 7.92-7.84 (m, 2H, H-4’ 2-FPh, H-9), 8.42 (d, 1H, *J=*7.9, H-11), 10.12 (s, 1H, -C(O)NHPh-F-2), 13.14 (s, 1H, -C(O)NH); ^13^C-NMR (100 MHz) δ: 18.20 (CH_3_), 35.28 (-S*C*H_2_C(O)NH-Thiadiazole), 38.12 (-S*C*H_2_C(O)NHPh-F-2), 116.01 (3’ 2-FPh), 118.62 (6’ 2-FPh), 124.19 (11a), 124.90 (8), 126.01 (5’ 2-FPh), 126.26 (1’ 2-FPh), 126.50 (10), 128.60 (11), 136.04 (4’ 2-FPh), 144.06 (9), 151.97 (3), 152.89 (11b), 154.19 (2’ 2-FPh), 154.85 (5’ Thiadiazole), 155.28 (7a), 158.78 (6), 159.76 (-C(O)NHPh), 161.02 (2), 166.60 (2’ Thiadiazole), 167.65 (CONH); EI-MS, *m/z* (I_rel_, %) = 354 (2.0), 285 (22.5), 244 (22.0), 243 (6.5), 217 (9.9), 204 (5.9), 203 (40.7), 200 (6.4), 186 (10.4), 185 (18.8), 176 (5.8), 175 (5.2), 174 (40.3), 171 (17.8), 170 (21.3), 161 (19.2), 160 (7.6), 153 (8.5), 148 (9.6), 147 (18.0), 146 (5.3), 145 (45.6), 144 (17.8), 143 (30.6), 142 (12.6), 138 (5.7), 137 (22.9), 136 (10.1), 135 (6.3), 134 (11.9), 129 (11.7), 124 (22.4), 122 (9.5), 118 (5.2), 117 (15.5), 116 (16.3), 115 (9.5), 114 (27.1), 112 (8.4), 111 (100.0), 110 (30.5), 109 (32.6), 108 (7.6), 103 (7.8), 102 (42.7), 100 (5.2), 98 (8.6), 95 (10.8), 91 (11.6), 90 (21.2), 89 (6.9), 88 (5.5), 86 (8.0), 85 (5.7), 84 (22.0), 83 (68.0), 82 (16.1), 81 (8.3), 78 (8.8), 77 (30.8), 76 (36.4),74 (11.3), 72 (6.8), 70 (28.0), 69 (10.5), 65 (5.5), 64 (22.1), 63 (24.1), 62 (11.8), 61 (5.9), 60 (34.1), 59 (13.6), 58 (7.4), 57 (23.8), 56 (13.2), 55 (6.3), 54 (6.3), 53 (5.0), 52 (12.0), 51 (13.7), 50 (11.8), 47 (18.6), 46 (35.4), 45 (32.8), 44 (17.1), 43 (16.9), 42 (15.7), 41 (15.6), 40 (10.6); LC-MS (APCI), *m/z* = 569 [M+1], 570 [M+2]; Anal. calcd. for C_23_H_17_FN_8_O_3_S_3_: C, 48.58; H, 3.01; N, 19.71; S, 16.92; Found: C, 48.57; H, 3.03; N, 19.70; S, 16.90.

#### N-[5-({2-[(2-Fluorophenyl)amino]-2-oxoethyl}thio)-1,3,4-thiadiazol-2-yl]-2-[(2-oxo-3-phenyl-2H-[1,2,4]triazino[2,3-c]quinazolin-6-yl)thio]acetamide (**6.3**)

Yield: 83.4%; M.p. 248–250°C; IR (cm^−1^): 3322, 3302, 3057, 2977, 2916, 2702, 1715, 1681, 1657, 1620, 1584, 1561, 1531, 1502, 1486, 1468, 1454, 1403, 1387, 1366, 1326, 1284, 1257, 1247, 1195, 1156, 1101, 1081, 1060, 1034, 1003, 992, 964, 942, 889, 852, 813, 771, 747, 688, 654, 638, 621; ^1^H-NMR (400 MHz) δ: 4.27 (s, 2H, -S-C*H*_2_-C(O)NHPh-F-2), 4.38 (s, 2H, -S-C*H*_2_-), 7.18-7.13 (m, 2H, H-3’, 6’ 2-FPh), 7.25 (t, 1H, *J=*7.3, H-5’, 2-F-Ph), 7.70-7.50 (m, 5H, H-3’, 4’, 5’ 3-Ph; H-8, H-10), 7.88 (t, 1H, H-4’ 2-F-Ph), 7.93 (t, 1H, *J=*7.7, H-9), 8.29 (d, 2H, *J=*7.3, H-2’, 6’ 3-Ph), 8.47 (d, 1H, *J=*7.9, H-11), 10.12 (s, 1H, -C(O)NHPh-F-2), 13.16 (s, 1H, -C(O)NH); ^13^C-NMR (100 MHz) δ: 35.23 (-S*C*H_2_C(O)NH-Thiadiazol), 38.13 (-S*C*H_2_C(O)NH-Ph-F-2), 115.94 (3’ 2-FPh), 118.40 (11a), 124.91 (6’ 2-FPh), 125.96 (5’ 2-FPh), 124.19 (10), 126.02 (8), 126.13 (1’ 2-FPh), 126.31 (11), 126.63 (4’ Ph), 128.21 (1’ Ph), 128.95 (3’, 5’ Ph), 129.84 (2’, 6’ Ph), 132.06 (4’ 2-FPh), 136.15 (9), 144.04 (3), 149.74 (11b), 151.08 (2’ 2-FPh), 154.48 (5’ Thiadiazol), 154.84 (7a), 158.84 (6), 159.68 (-C(O)NHPh-F-2), 160.15 (2), 166.59 (2’ Thiadiazol), 167.60 (CONH); LC-MS (APCI), *m/z* = 631 [M+1], 632 [M+2]; Anal. calcd. for C_28_H_19_FN_8_O_3_S_3_: C, 53.32; H, 3.04; N, 17.77; S, 15.25; Found: C, 53.35; H, 3.06; N, 17.79; S, 15.28.

#### N-[5-({2-[(3-Chlorophenyl)amino]-2-oxoethyl}thio)-1,3,4-thiadiazol-2-yl]-2-[(3-methyl-2-oxo-2H-[1,2,4]triazino[2,3-c]quinazolin-6-yl)thio]acetamide (**6.4**)

Yield: 94.8%; M.p. 243–245°C; IR (cm^−1^): 3262, 3185, 3129, 3087, 2911, 2837, 2714, 1690, 1650, 1583, 1494, 1467, 1417, 1395, 1362, 1321, 1284, 1268, 1236, 1173, 1138, 1103, 1071, 1048, 995, 958, 878, 827, 766, 712, 682, 658, 633; ^1^H-NMR (400 MHz) δ: 2.38 (s, 3H, 3-C*H*_3_), 4.23 (s, 2H, -S-C*H*_2_-C(O)NHPh-F-2), 4.34 (s, 2H, -S-C*H*_2_-), 7.12 (s, 1H, *J^3^*=7.7, *J^4^*=1.2, H-4’ 3-ClPh), 7.33 (t, 1H, *J=*8.1, H-5’ 3-Cl-Ph), 7.46 (d, *J=*7.9, H-6’ 3-ClPh), 7.55 (d, 1H, *J=*7.9, H-8), 7.63 (t, 1H, *J=*7.7, H-10), 7.78 (s, 1H, H-2’ 3-ClPh), 7.91 (t, 1H, *J^3^*=7.7, *J^4^*=1.2, H-9), 8.42 (d, 1H, *J^3^*=7.9, *J^4^*=1.2, H-11), 10.53 (s, 1H, -C(O)NHPh-C1-3), 13.15 (s, 1H, -C(O)NH); LC-MS (APCI), *m/z* = 584 [M-1], 587 [M+2]; Anal. calcd. for C_23_H_17_ClN_8_O_3_S_3_: C, 47.22; H, 2.93; Cl, 6.06; N, 19.15; S, 16.44; Found: C, 47.23; H, 2.95; Cl, 6.08; N, 19.15; S, 16.45.

#### N-[5-({2-[(3-Chlorophenyl)amino]-2-oxoethyl}thio)-1,3,4-thiadiazol-2-yl]-2-[(2-oxo-3-phenyl-2H-[1,2,4]triazino[2,3-c]quinazolin-6-yl)thio]acetamide (**6.5**)

Yield: 89.6%; M.p. 266–269°C; IR (cm^−1^): 3292, 3197, 3119, 3066, 3003, 2964, 2929, 1677, 1656, 1587, 1565, 1537, 1503, 1479, 1443, 1425, 1402, 1380, 1336, 1312, 1287, 1265, 1249, 1176, 1163, 1141, 1090, 1075, 1057, 1030, 1001, 989, 970, 940, 916, 894, 862, 846, 813, 803, 777, 755, 736, 687, 679, 654, 640, 621; ^1^H-NMR (400 MHz) δ: 4.21 (s, 2H, -S-CH_2_-C(O)NHPh-F-2), 4.37 (s, 2H, -S-CH_2_-), 7.13 (s, 1H, J=7.7, H-4’ 3-ClPh), 7.34 (t, 1H, J=8.1, H-5’ 3-Cl-Ph), 7.41 (d, J=7.9, H-6’ 3-ClPh), 7.70-7.57 (m, 5H, H-3’, 4’, 5’ 3-Ph; H-8, H-10), 7.77 (s, 1H, H-2’ 3-ClPh), 7.93 (t, 1H, J=7.7, H-9), 8.29 (d, 2H, J=7.3, H-2’, 6’ 3-Ph), 8.47 (d, 1H, J=7.9, H-11), 10.53 (s, 1H, -C(O)NHPh-Cl-3), 13.15 (s, 1H, -C(O)NH); LC-MS (APCI), m/z = 646 [M-1], 647 [M], 648 [M+1], 651 [M+4]; Anal. calcd. for C_28_H_19_ClN_8_O_3_S_3_: C, 51.97; H, 2.96; Cl, 5.48; N, 17.31; S, 14.86; Found: C, 51.96; H, 2.93; Cl, 5.47; N, 17.29; S, 14.85.

### Biological assay

#### Bioluminescence inhibition test

The marine luminescent bacteria *Photobacterium leiognathi* strain Sh1, isolated from the Azov Sea Shrimp, were used for the bioluminescence analysis [25, 26]. Bacteria were cultivated on a nutrient environment containing (g/L): pepton – 5, yeast extract – 1.5, meat extract – 1.5, sodium chloride – 30, pH = 7.4. In the acute action test (inhibiting the luminescence of bacteria), bacteria were diluted with the 3% sodium chloride solution up to a concentration of 10^5^ cells/mL. 5–50 mg/mL of the studied substances suspended in DMSO were mixed with 1 mL of the diluted bacterial suspension. Vials were incubating for 10 min at 25°C, and then the intensity of bioluminescence was measured as the percent (%) relative to the controls, which were prepared without the studied compounds. In the chronic action test (inhibiting growth and luminescence of bacteria), the growth medium was added for potential breeding in a ratio of 1:50 and the mix was incubated for 16–18 h at 30°C, whereupon the intensity of bioluminescence was measured the same way as in the acute action testing. Tetracycline was used as a reference. The bacterial luminescence was measured with a Bioluminometer BLM-8801 («Science», Krasnoyarsk, Russia).

#### Cytotoxic activity against malignant human tumor cells

The primary anticancer assay was performed at the human tumor cell lines panel derived from nine neoplastic diseases, in accordance with the protocol of the Drug Evaluation Branch, National Cancer Institute, Bethesda [27–30]. Tested compounds were added to the culture at a single concentration (10^−5^ M) and the cultures were incubated for 48 h. End point determinations were made with a protein binding dye, sulforhodamine B (SRB). Results for each tested compound were reported as the percent of growth of the treated cells when compared to the untreated control cells. The percentage growth was evaluated spectrophotometrically versus controls not treated with the test agents. The cytotoxic and/or growth inhibitory effects of the most active selected compounds were tested *in vitro* against the full panel of about 60 human tumor cell lines at 10-fold dilutions of five concentrations ranging from 10^−4^ to 10^−8^ M. A 48-h continuous drug exposure protocol was followed and an SRB protein assay was used to estimate cell viability or growth. Using the seven absorbance measurements [time zero, (T_z_), control growth in the absence of the drug (C), and test growth in the presence of the drug at the five concentration levels (T_i_)], the percentage growth was calculated at each of the drug concentrations levels. Percentage growth inhibition was calculated as:

$$[({\text{T}}_{\text{i}}-{\text{T}}_{\text{z}})/(\text{C}-{\text{T}}_{\text{z}})]\times 100\;{\text{for}\;\text{concentrations}\;\text{for}\;\text{which}\;\text{T}}_{\text{i}}\geq {\text{T}}_{\text{z}},$$

$$[({\text{T}}_{\text{i}}-{\text{T}}_{\text{z}})]/{\text{T}}_{\text{z}}]\times 100\;{\text{for}\;\text{concentrations}\;\text{for}\;\text{which}\;\text{T}}_{\text{i}}{<\text{T}}_{\text{z}}.$$

Three dose response parameters were calculated for each compound. Growth inhibition of 50% (GI_50_) was calculated from [(T_i_ − T^z^)/(C − T_z_)] × 100 = 50, which is the drug concentration resulting in a 50% lower net protein increase in the treated cells (measured by SRB staining) as compared to the net protein increase seen in the control cells. The drug concentration resulting in total growth inhibition (TGI) was calculated from T_i_ = T_z_. The LC_50_ (concentration of the drug resulting in a 50% reduction in the measured protein at the end of the drug treatment as compared to that at the beginning) indicating a net loss of cells following treatment, was calculated from [(T_i_ − T_z_)/T_z_] ×100 = -50. Values were calculated for each of these three parameters if the level of activity was reached; however, if the effect was not reached or was exceeded, the value for that parameter was expressed as greater or less than the maximum or minimum concentration tested. The log GI_50_, log TGI, and log LC_50_ were then determined, defined as the mean of the logs of the individual GI_50_, TGI, and LC_50_ values. The lowest values were obtained with the most sensitive cell lines.

## Figures and Tables

**Sch. 1.**
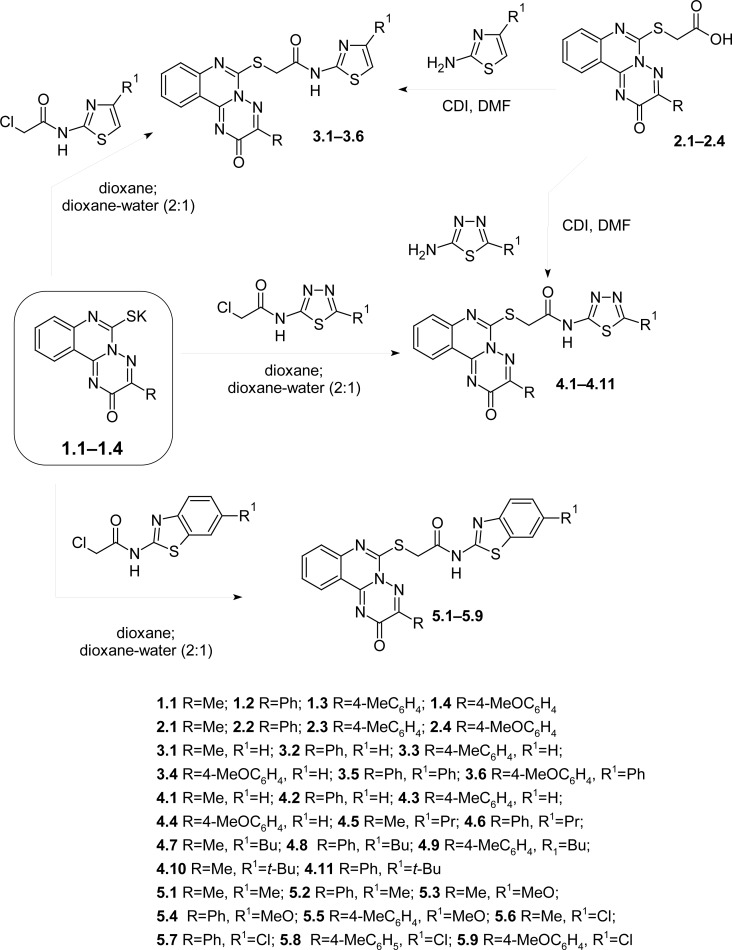
The approaches to the synthesis of *N*-*R*-2-[(3-R-2-oxo-2*H*-[1,2,4]triazino [2,3-*c*]quinazolin-6-yl)thio]acetamides with thiazole and thiadiazole fragments.

**Sch. 2.**
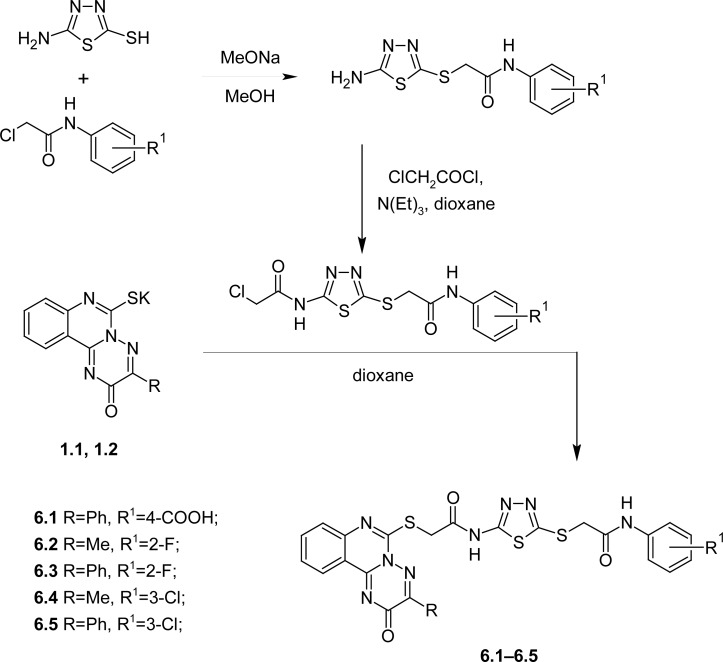
Synthesis of *N*-R-2-[(3-R-2-oxo-2*H*-[1,2,4]triazino[2,3-*c*]quinazolin-6-yl)thio]acetamides with 2-S-substituted 2-thiothiadiazole fragments.

**Tab. 1.** Bioluminescence inhibiting assay data, %

**Compd.**	**Control**	**Acute action test (mg/mL)**			**Chronic action test (mg/mL)**		

		**0.025**	**0.1**	**0.25**	**0.025**	**0.1**	**0.25**
DMSO	100.00	100.00^a^	100.00	100.00	100.00	100.00	100.00
3.1	100.00	114.80	111.50	122.60	62.50	22.20	33.30
3.2	100.00	115.00	100.00	157.10	93.30	90.90	75.00
3.3	100.00	103.60	127.30	109.10	130.80	16.92	2.12
3.4	100.00	106.30	125.00	133.30	79.20	102.30	60.00
3.5	100.00	100.00	101.10	97.96	16.67	10.67	1.00
3.6	100.00	75.00	79.29	62.50	150.90	150.90	7.55
4.1	100.00	125.00	100.00	133.30	66.00	163.60	85.50
4.2	100.00	100.00	105.00	133.30	1.70	0.50	0.40
4.3	100.00	90.00	90.00	100.00	133.30	50.00	16.67
4.4	100.00	102.20	125.20	179.10	64.80	52.00	57.10
4.5	100.00	100.00	93.75	62.50	105.90	64.71	17.65
4.6	100.00	81.50	100.00	130.30	72.00	64.00	8.00
4.7	100.00	100.0	95.24	78.57	105.90	107.30	102.94
4.8	100.00	92.30	106.00	130.30	125.30	166.40	160.00
4.9	100.00	81.50	90.00	85.20	93.30	166.40	80.00
4.10	100.00	98.85	94.25	2.30	100.0	53.33	18.33
4.11	100.00	93.75	88.54	83.33	166.70	183.30	133.33
5.1	100.00	131.40	115.90	119.40	16.70	2.40	0.60
5.2	100.00	66.70	62.50	71.70	118.20	53.80	100.00
5.3	100.00	75.71	75.71	5.71	103.20	84.21	31.58
5.4	100.00	50.00	49.00	44.30	100.00	21.30	17.5
5.5	100.00	84.44	80.00	77.78	31.25	30.00	6.25
5.6	100.00	103.1	86.15	92.31	94.62	64.52	1.08
5.7	100.00	91.70	89.30	150.00	46.50	93.30	150.00
5.8	100.00	122.50	83.80	125.00	184.90	152.30	6.40
5.9	100.00	96.92	103.10	72.31	88.89	83.33	71.11
6.2	100.00	82.22	75.56	57.78	95.71	95.71	85.71
6.3	100.00	103.30	27.17	3.26	93.33	100.00	133.33
6.4	100.00	100.00	83.33	68.75	187.90	175.80	181.82
6.5	100.00	75.44	78.95	78.95	43.10	37.93	6.90
Tetracycline	100.00	80.70	9.10	0.00	0.00	0.00	0.00

a Bioluminescence intensity %.

**Tab. 2.** Percentage of *in vitro* tumor cell lines growth at 10 μM for compounds

**Cpd.**	**Mean**	**Range of**	**Most sensitive cell line growth, %^a^**

** growth %**		
3.1	29.76	−30.75–75.84	13.10 (CCRF-CEM/L), 20.51 (HL-60(TB)/L), 19.99 (K-562/L), 36.69 (MOLT-4/L), 22.37 (RPMI-8226/L), 7.45 (SR/L), 36.59 (NCI-H23/nscLC), 35.65 (NCI-H322M/nscLC), 19.20 (NCI-H460/nscLC), 20.83 (NCI-H522/nscLC), 11.77 (COLO 205/CC), 33.70 (HCT-116/CC), 31.24 (HCT-15/CC), 26.28 (KM12/CC), 17.54 (SW-620/CC), −30.75 (SF-295/CNSC), 0.96 (SF-539/CNSC), 37.69 (SNB-19/CNSC), 8.26 (SNB-75/CNSC), 30.68 (U251/CNSC), 34.39 (LOX IMVI/M), 55.31 (MALME-3M/M), 29.29 (M14/M), −27.89 (MDA-MB-435/M), 5.27 (SK-MEL-2/M), 9.51 (SK-MEL-5/M), 39.79 (UACC-257/M), 27.77 (UACC-62/M), 33.99 (IGROV1/OV), 1.13 (OVCAR-3/OV), 33.74 (OVCAR-8/OV), 21.74 (NCI/ADR-RES/OV), 28.77 (SK-OV-3/OV), 10.07 (CAKI-1/RC), 3.96 (RXF 393/RC), 41.37 (SN12C/RC), 37.81 (UO-31/RC), 23.11 (PC-3/PC), 32.38 (DU-145/PC), 14.85 (MCF7/BC), 44.10 (BT-549/BC), −3.37 (MDA-MB-468/BC)
3.2	51.53	−10.00–101.82	0.84 (CCRF-CEM/L), −7.51 (HOP-92/nsscLC), 3.57 (SNB-75/CNSC), 1.73 (LOX IMVI/M), 20.30 (MALME-3M/M), 6.05 (SK-MEL-28/M), 24.06 (OVCAR-3/OV), −10.00 (OVCAR-4/OV), 6.47 (OVCAR-8/OV)
3.4	91.57	28.74–116.21	28.74 (CCRF-CEM/L)
4.1	97.74	−33.94–124.48	−33.94 (CCRF-CEM/L), 72.33 (MOLT-4/L)
4.2	98.86	10.72–123.07	10.72 (CCRF-CEM/L)
4.4	101.17	−24.53–123.74	−24.53 (CCRF-CEM/L), 55.76 (MOLT-4/L)
4.9	85.43	37.80–122.83	66.93 (CCRF-CEM/L), 62.97 (MOLT-4/L), 56.31 (SR/L), 59.58 (HCT-116/CC), 37.80 (SW-620/CC), 62.55 (U251/CNSC), 50.07 (LOX IMVI/M), 62.00 (OVCAR-8/OV),56.12 (MDA-MB-231/ATCC/BC)
5.1	82.03	34.38–114.27	34.38 (NCI-H522/nscLC)
5.4	92.98	49.44–121.56	49.44 (NCI-H522/nscLC)
5.7	90.48	−13.28–145.89	−13.28 (CCRF-CEM/L)
5.8	92.07	18.56–127.87	31.38 (CCRF-CEM/L), 18.56 (OVCAR-8/OV)
6.4	102.03	15.40–131.22	15.40 (CCRF-CEM/L)
6.5	44.20	−65.63–110.88	−34.06 (CCRF-CEM/L), 24.09 (NCI-H460/nscLC), 25.42 (NCI-H522/nscLC), 22.87 (HCT-116/CC), 1.68 (KM12/CC),10.61 (SW-620/CC), 18.11 (SF-539/CNSC), 15.71 (SNB-75/CNSC), 17.91 (U251/CNSC), 0.55 (LOX IMVI/M), −20.25 (MALME-3M/M), 28.31 (MDA-MB-435/M), −31.61 (SK-MEL-28/M), 6.64 (SK-MEL-5/M), 7.21 (IGROV1/OV), −38.44 (OVCAR-3/OV), −36.94 (OVCAR-4/OV), −65.63 (OVCAR-8/OV), 20.18 (MCF7/BC), 27.44 (MDA-MB-231/ATCC/BC)

a L – leukemia, nscLC – non-small cell lung cancer, ColC – colon cancer, CNSC – CNS cancer, M – melanoma, OV– ovarian cancer, RC – renal cancer, PC – prostate cancer, BC – breast cancer.

**Tab. 3.** The influence of compounds on the growth of individual tumor cell lines (GI_50_≤ 1.00 μM)

**Disease**	**Cell line**	**Cpd. 3.1**		**Cpd. 3.2**		**Cpd. 6.5**	

		**GI_50_**	**TGI**	**GI_50_**	**TGI**	**GI_50_**	**TGI**
MG_MID		6.47	83.43	19.69	46.21	11.42	55.96

Leukemia	CCRF-CEM	3.00	>100	>100	>100	9.37	>100
	K-562	0.34	>100	9.41	>100	8.40	>100
	MOLT-4	3.39	>100	>100	>100	30.00	>100
	RPMI-8226	32.10	>100	7.06	>100	26.70	>100
	SR	0.38	>100	>100	>100	>100	>100

NSC lung cancer	A549/ATCC	5.89	>100	2.45	7.16	3.12	10.00
	EKVX	–	–	8.94	>100	12.30	63.00
	HOP-62	3.48	35.40	2.91	12.30	12.00	26.20
	HOP-92	–	–	2.04	4.92	2.78	11.30
	NCI-H226	18.20	>100	2.54	8.35	3.61	15.90
	NCI-H23	2.96	>100	10.00	>100	3.36	15.20
	NCI-H322M	8.17	>100	8.21	81.50	21.90	>100
	NCI-H460	0.68	>100	3.03	8.56	3.45	13.30
	NCI-H522	0.22	1.30	5.15	33.50	10.10	29.50

Colon cancer	COLO 205	0.45	5.14	3.70	87.30	6.56	21.50
	HCC-2998	–	–	7.26	>100	33.90	>100
	HCT-116	0.44	>100	2.21	5.24	2.78	10.30
	HCT-15	0.69	>100	7.55	>100	>100	>100
	HT29	0.41	>100	3.71	17.30	3.40	10.20
	KM12	0.49	>100	5.81	>100	3.20	11.70
	SW-620	0.54	>100	4.92	>100	4.20	37.20

CNS cancer	SF-268	6.50	>100	5.76	>100	4.90	20.90
	SF-295	–	–	2.73	10.80	10.30	31.20
	SF-539	1.20	13.40	5.18	68.00	1.99	5.34
	SNB-19	7.61	>100	10.30	>100	9.17	26.40
	SNB-75	2.24	18.50	2.00	4.47	5.40	18.80
	U251	2.45	>100	3.20	16.10	3.44	12.90

Melanoma	LOX IMVI	4.22	>100	8.69	>100	7.36	23.20
	MALME-3M	0.53	>100	2.97	9.92	4.44	17.50
	M14	0.56	>100	4.98	91.90	6.18	22.10
	MDA-MB-435	–	–	4.35	>100	5.63	37.80
	SK-MEL-2	13.50	>100	4.86	19.30	12.10	37.70
	SK-MEL-28	4.40	>100	3.39	10.80	4.12	14.50
	SK-MEL-5	0.48	>100	3.30	17.80	3.14	13.40
	UACC-257	3.12	>100	3.81	50.90	14.00	>100
	UACC-62	0.58	>100	6.02	>100	11.80	99.00

Ovarian cancer	IGROV1	0.81	>100	5.47	73.20	4.13	16.20
	OVCAR-3	0.26	0.73	2.99	11.40	2.19	4.95
	OVCAR-4	–	–	1.96	4.05	2.11	5.00
	OVCAR-5	5.01	>100	8.00	>100	19.90	>100
	OVCAR-8	2.97	>100	4.75	>100	3.16	10.10
	NCI/ADR-RES	0.72	>100	6.14	>100	>100	>100
	SK-OV-3	2.49	41.90	2.15	6.11	11.20	25.90

Renal cancer	786-0	1.78	>100	5.17	21.50	42.10	>100
	A498	0.68	8.47	2.34	24.80	15.30	55.10
	ACHN	14.90	>100	3.14	21.10	6.26	>100
	CAKI-1	–	–	2.74	11.90	>100	>100
	RXF 393	0.69	3.97	2.24	6.33	11.10	25.60
	SN12C	6.98	>100	6.43	>100	8.08	36.60
	UO-31	1.84	>100	2.52	13.80	>100	>100

Prostate cancer	PC-3	3.98	>100	3.21	15.40	2.64	7.70
	DU-145	1.72	>100	3.61	27.60	11.10	72.20

Breast cancer	MCF7	0.48	>100	4.85	>100	4.34	16.50
	MDA-MB-231/ATCC	14.40	>100	4.95	34.20	4.91	21.50
	BT-549	0.93	>100	9.48	86.50	11.60	33.80
	T-47D	46.00	>100	7.65	97.90	8.33	42.00
	MDA-MB-468	0.31	9.31	3.85	23.60	12.00	51.20

**Tab. 4.** Anticancer selectivity pattern of the most active compounds at the GI_50_ (C, μM) and TGI (C, μM) levels

**Cpd.**	**Parameters**	**Disease**								
		**L**	**NSCLC**	**ColC**	**CNSC**	**M**	**OV**	**RC**	**PC**	**BC**
3.1	GI_50_	7.84	5.66	7.55	4.60	6.93	2.05	7.15	2.85	12.42
	SI^*^	0.83	1.14	0.86	1.41	0.93	3.16	0.90	2.27	0.52
	TGI	>100	76.67	86.45	66.38	>100	73.77	68.74	>100	81.86
	SI^**^	0.83	1.08	0.97	1.26	0.83	1.13	1.21	0.83	1.02
3.2	GI_50_	69.41	5.03	5.02	4.86	4.71	4.49	3.51	3.41	6.16
	SI^*^	0.16	2.27	2.28	2.35	2.42	2.54	3.25	3.35	1.85
	TGI	>100	39.59	72.83	49.90	56.62	56.39	28.49	21.50	68.44
	SI^**^	0.56	1.41	0.77	1.14	0.99	0.99	1.96	2.60	0.82
6.5	GI_50_	45.75	8.07	22.01	5.87	7.64	20.38	48.44	6.87	8.24
	SI^*^	0.43	2.44	0.89	3.35	2.58	0.97	0.41	2.87	2.39
	TGI	>100	31.60	41.56	19.26	40.58	37.45	73.90	39.95	33.00
	SI^**^	0.46	1.46	1.11	2.40	1.14	1.23	0.63	1.16	1.40

a Selectivity index at the GI_50_ (C, μM) level;

b Selectivity index at the TGI (C, μM) level.

L – leukemia, nscLC – non-small cell lung cancer, ColC – colon cancer, CNSC – CNS cancer, M – melanoma, OV– ovarian cancer, RC – renal cancer, PC – prostate cancer, BC – breast cancer.
